# The mechanochemical synthesis of polymers

**DOI:** 10.1039/d1cs01093j

**Published:** 2022-03-18

**Authors:** Annika Krusenbaum, Sven Grätz, Getinet Tamiru Tigineh, Lars Borchardt, Jeung Gon Kim

**Affiliations:** Anorganische Chemie I, Ruhr-Universität Bochum Universitätsstraße 150 44801 Bochum Germany lars.borchardt@rub.de; Department of Chemistry, Bahir Dar University Peda Street 07, PO Box 79 Bahir Dar Amhara Ethiopia; Department of Chemistry and Research Institute of Physics and Chemistry, Jeonbuk National University Jeon-Ju, Jeollabuk-do 54896 Republic of Korea jeunggonkim@jbnu.ac.kr

## Abstract

Mechanochemistry – the utilization of mechanical forces to induce chemical reactions – is a rarely considered tool for polymer synthesis. It offers numerous advantages such as reduced solvent consumption, accessibility of novel structures, and the avoidance of problems posed by low monomer solubility and fast precipitation. Consequently, the development of new high-performance materials based on mechanochemically synthesised polymers has drawn much interest, particularly from the perspective of green chemistry. This review covers the constructive mechanochemical synthesis of polymers, starting from early examples and progressing to the current state of the art while emphasising linear and porous polymers as well as post-polymerisation modifications.

## Abstract 2

Mechanochemistry enables solvent-free syntheses of even novel polymer structures without the need of monomer solubility.

## Graphical abstract

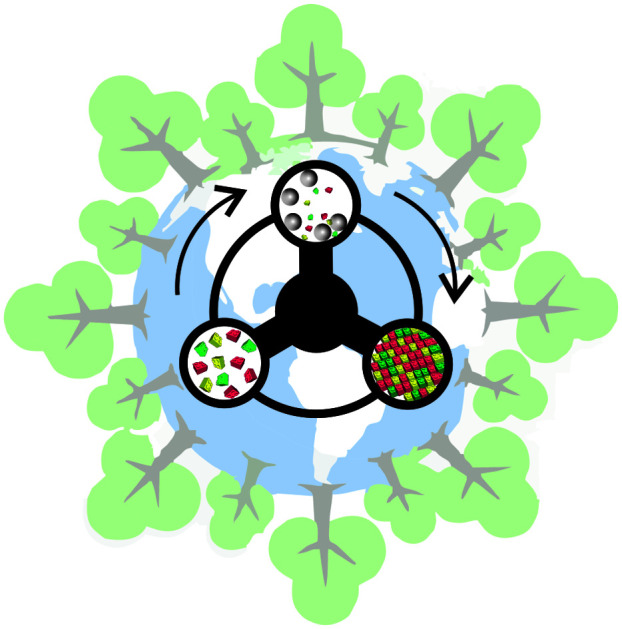

## Introduction

Mechanochemistry is a fast-growing area in chemistry. The efficient energy dispersion and mass transportation due to the use of mechanical forces has facilitated the elimination of solvents and warranted cleaner, faster, and more straightforward chemical syntheses than conventional solvent-based reactions.^1–4^ With many reported examples, mechanochemistry has become a reliable tool in synthesis. In particular, the application of mechanochemistry to the synthesis of molecules difficult or impossible to obtain by conventional methods has advanced many areas of chemistry,^5,6^ and mechanochemical activation has now been established as a promising technique alongside thermal-, photo-, and electroactivation.

Polymer mechanochemistry has a rich history. From the dawn of polymer science, chemical events related to mechanical actions such as the grinding, shearing, and stretching of polymer chains have been a topic of interest. In the 1930s, Staudinger and coworkers reported that the action of an external force on polymer chains induces their scission and thus reduces molecular weight, viscosity, and strength,^7–9^ defining polymer mechanochemistry as the science and engineering of chain breakage caused by the failure, fatigue, or abrasion of polymeric materials.^10^ Although the increasing understanding of destructive polymer mechanochemistry has inspired its numerous and diverse ‘smart’ applications,^11–13^ the development and applications of constructive monomer-to-polymer mechanochemistry lag behind.

The 100 year history of modern polymer science has witnessed the development and commercialisation of many new polymers,^14^ with the development and refinement of novel polymerisation techniques resulting in a previously unimaginable level of variety and precision. However, the underlying synthetic process has remained unchanged, *i.e.*, most polymerisations are still carried out in a fluidic phase using either a large excess of solvent or the melting of solid monomers upon excessive heating. This approach features the drawbacks of significant initial investment, high energy consumption, and problematic waste disposal/safety control^15,16^ and is barely applicable to the synthesis of polymers with limited solubility or miscibility.

Recently, mechanochemical polymer synthesis has drawn much attention in view of the inherent advantages of mechanochemistry such as greenness and access to novel reactivity.^15^ Additionally, solid-state mechanochemical polymer synthesis allows one to avoid solubilising group usage and low polymerisation degrees (DPs) caused by fast precipitation, *i.e.*, is well suited for the production of structures that cannot be accessed by solution processes.

The first mechanochemical polymerisation was demonstrated in 1959 by the Kargin group, who used vibrational ball milling to polymerise vinyl monomers.^17^ Several sporadic studies followed, including those of vinyl monomer polymerisation by Oprea in the 1980s^18^ and by Kuzuya in the 1990s.^19^ As mechanochemistry was not well established at that time, these studies did not receive deserved attention. In addition to the development of green and sustainable processes, mechanochemistry drew much interest at the beginning of the 21st century. The mechanochemical Gilch polymerisation of poly(phenylene vinylene) reported by Ravnsbæk and Swager in 2014 highlighted the potential of constructive polymer mechanochemistry.^20^ Since then, many research teams confirmed the suitability of mechanochemistry for the construction of one- to three-dimensional polymers *via* step-and chain-growth polymerisations.

The re-emergence of mechanochemistry within the last decade has inspired several reviews focusing on the broad topic of mechanochemistry and several subdisciplines.^21–26^

While the general discipline of mechanochemistry relies on the generation of reactive sites by either shearing, grinding, or stretching, the subdiscipline polymer mechanochemistry is nowadays divided into two different communities.^27,28^ Based on Staudinger's work on molecular weight reduction of polystyrene, the broad field of sonication and force spectroscopy emerged as a very important aspect of polymer mechanochemistry.^29^ The field expanded rapidly around the careful design and activation of mechanophores and has already been covered in several reviews.^29–35^

In contrast to this, we herein focus on the other part of polymer mechanochemistry, which comprises of mechanochemical polymerisation reactions induced by shearing and grinding during mortar-and-pestle synthesis, high-speed ball milling and reactive extrusion. To the best of our knowledge, this aspect of polymer mechanochemistry has not been addressed in a review by now, although it is recently gaining huge attention by the chemical community.

This review provides insights into the constructive mechanochemical synthesis of polymers, starting with the basics of mechanochemistry and then describing the syntheses of linear and porous polymers in separate chapters. We believe that this structure is convenient, as the related articles are very different in scope and originate from different communities. Nevertheless, we aim to bring them together within this review, showcasing the underlying similarities.

## Mechanochemistry

It was Matthew Carey Lea in 1892 who postulated that mechanochemical reactions can be fundamentally different to thermochemical ones and it was Wilhelm Ostwald who coined the term “mechanochemistry” in 1907 as distinct branch of chemistry alongside electrochemistry, photochemistry, and thermochemistry.^36,37^ Since these days, the field of mechanochemistry has constantly expanded, from Staudinger's pioneering work on polymer mechanochemistry in the 1930s^8^ over different reports of supramolecular mechanochemistry in the 1980s^38,39^ and solid–solid organic synthesis strategies in the 1990s.^40–42^

Today, the IUPAC defines a mechanochemical reaction as a chemical reaction induced by the direct absorption of mechanical energy.^27^ Despite the rapid expansion of shearing- and grinding-induced mechanochemistry and the routine publication of new synthetic procedures for various products, the mechanism at microscopic and molecular levels, however, remains unclear. High-speed ball mills and reactive extruders are often described as ‘black boxes’, as the chemical processes therein are shielded by enclosures, which are generally made of metals or ceramics.^43^ This leads to one of the major obstacles of mechanochemistry, which is the lack of a valid theory describing the ongoing reactions entirely, while being ubiquitously applicable towards various synthetic protocols. As of now, three different theories, namely the hot-spot theory,^44,45^ the magma-plasma model,^46,47^ and the pseudo-fluid model,^48^ are known for mechanochemistry, each capable to describe certain phenomena of various subdisciplines.^49,50^ Unfortunately, they all fail to explain mechanochemical polymerization reactions thoroughly. Any theory, developed in the future, should take the following important observations into consideration:

• Milling achieves the intimate mixing of substrates on the sub-micrometre scale, constantly creates fresh reactive surface, and thus decreases diffusion limitations.^51^

• Mechanical stress generates heat^52,53^ and minor temperature increases can lead to huge increases of the mechanochemical reaction rate.^54–57^

• The milling parameters play a major role in enabling and controlling mechanochemical reactions.^56,58–60^

• The collision inside the vessel can lead to the generation of radicals.^61,62^

## Mechanochemical reactors

The simplest mechanochemical method has been (and still is) the utilisation of the mortar and pestle to induce chemical reactions between solid materials. Although this protocol is still used for the generation of polymers,^63,64^ the method itself has certain limitations. Owing to the size of the mortar, the typical synthesis batch is in the milligram to lower gram range and cannot be upscaled to meet the needs of industrial applications.^65^ Additionally, the synthesis time usually does not exceed several hours and is mainly limited by the human operator, while grinding itself is utterly slow. The most important drawback is the inhomogeneous energy input. In particular, with longer grinding times, the energy input continuously decreases, which makes the entire process barely reproducible.^65^ Likewise, reproducibility depends on the experimenter, as Stolle and co-workers have shown.^66^

These drawbacks have been addressed by the invention of automated grinding devices. An important step in this direction was accomplished by the development of a motorised mortar-and-pestle setup by Retsch in 1923.^67^ Automated ball mills designed for size reduction, such as tumbler mills (invented in 1870) and vibrational ball mills (invented in 1930), are also used for mechanochemical syntheses.^68^

**Fig. 1:**
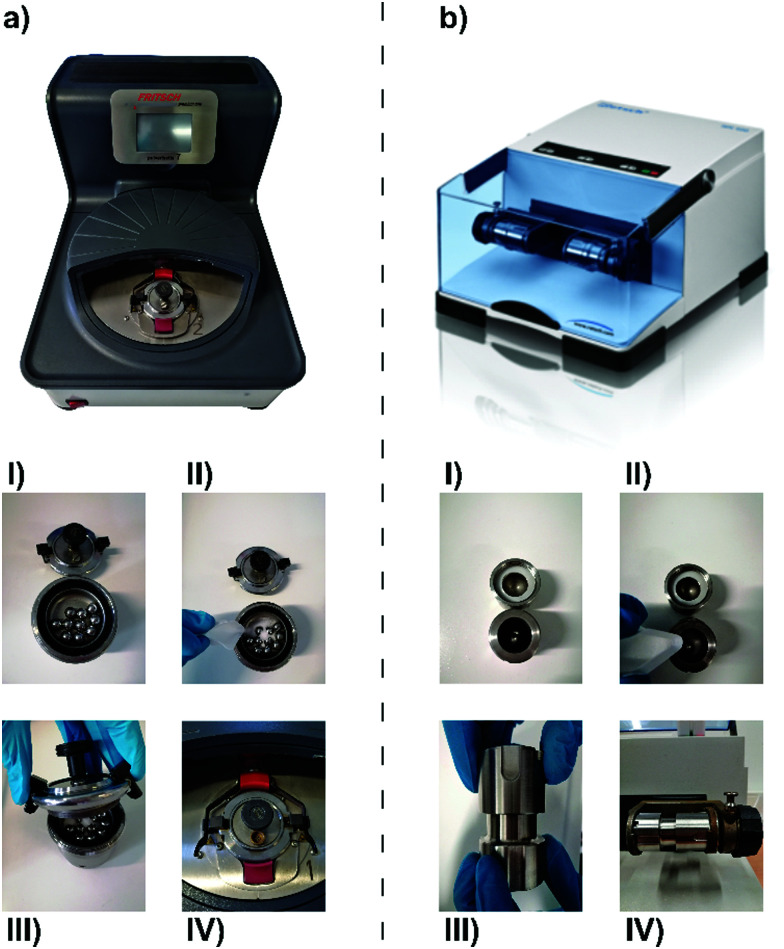
Upper part: Examples of (a) a planetary ball mill (Fritsch planetary ball mill P7) and (b) a vibrational ball mill (Retsch mixer mill MM400). Lower part: Procedure of milling jar filling for (left) a planetary ball mill and (right) a vibrational ball mill. (I) The empty milling jars are equipped with a seal and milling balls. (II) The mill charge is added to the jar. (III) Milling jars are properly closed. (IV) Implementation of the milling jars in the respective ball mill.

A tumbler mill is a horizontally mounted grinding drum operated with the aid of gravity, as the milling bodies (either balls or rods) drop from a certain height onto the vessel wall and the reaction mixture to induce a reaction between the particles. Tumbler mills have not been developed for chemical reactions in the laboratory, as laboratory-scale applications require the mill and, hence, the dropping height and impact energy, to be much smaller. To counteract this, in 1961, Fritsch placed four vertically mounted tumblers onto a counter-rotating sun disc to develop the first planetary ball mill (a lab-scale state-of-the-art planetary ball mill is shown in Fig. 1a).^67^

Because of this setup, the grinding media are accelerated by rotation, which leads to friction and collisions between the milling balls, the jar, and the reactants,^69,70^ and the kinetic energy is transferred to the reaction particles to induce bond breakage, bond formation, and size reduction. Furthermore, the kinetic energy is transformed into thermal energy, which results in a continuous temperature rise inside the milling jar.^22,71,72^

Another ball mill ubiquitously used for lab-scale mechanochemical synthesis is the vibrational ball mill, which is usually equipped with two to six milling jars (Fig. 1b). During the milling process, the jar follows a horizontal, vertical, or elliptical course at high frequencies.^73^ Owing to the inertia of the milling material and the mill charge, the fast changes of direction during vibration result in collisions and friction of the interior with itself and the vessel walls. The milling frequency and the horizontal/vertical deflection are directly linked to impact energy.^43^ In contrast to planetary ball milling, which is dominated by shear forces, vibrational ball milling is dominated by impact forces, as in the latter case, the sample is exposed to short pulses of mechanical energy.^65^

The design of high-speed ball mills has significantly evolved during the development of mechanochemistry. As mentioned previously, the mechanical energy is partially converted into thermal energy to heat up the milling jar. Consequently, energy management lacks the controllability required for upscaling to industrial batch sizes of kilograms or even tons.^69^ Retsch GmbH invented a cryomill, *i.e.*, a vibrational ball mill equipped with an internal cooling system. Although this mill was originally developed for polymer milling, chemists have adapted the underlying principle to conduct reactions at constant temperatures. In this case, impact energy can be considered to be independent of thermal energy, which delivers new insights into the underlying mechanochemical mechanism.^74,75^ Concurrently, a mill setup with electric cuffs enables the heating of steel milling jars during synthesis, which is especially useful for temperature-dependent reactions such as the mechanochemical generation of microporous polymers^53^ and metal–organic frameworks^57^ or for obtaining fundamental insights into the temperature dependence of mechanochemical reactions.^76,77^ These latest developments make temperature-regulating milling equipment more accessible for mechanochemists around the globe.

The invention of transparent milling vessels made of quartz, sapphire, poly(perfluoroalkoxy alkane)s or poly(methyl methacrylate) allows the simultaneous mechano- and photochemical acceleration in a photoreactor^78–81^ as well as *in situ* (*e.g.*, Raman spectroscopic) measurements, thus providing access to non-isolatable reactive intermediates.^82–84^ Likewise, *in situ* powder X-ray diffraction enables the real-time detection of crystalline materials and their transformations during milling.^78,85–87^ Recently, Emmerling and Van Wüllen reported the first monitoring of a mechanochemical reaction by *in situ* solid-state NMR spectroscopy.^88^ Specifically, the ^31^P spectrum with and without proton decoupling was monitored in a self-made miniaturised ball mill on an NMR sample head. Another important *in situ* technique is the simultaneous measurement of temperature and pressure using a gas pressure and temperature measurement system. In particular, for the synthesis of porous polymers, the recording of pressure build-up can assist mechanism elucidation.^53,89^

Although planetary and vibrational ball mills are highly efficient for polymer synthesis,^90,91^ their low material throughput is a definite drawback. Typically, milling jars have a volume of 1–500 mL, which limits the batch sizes to several grams, as jars are usually filled to one-third with milling balls, one-third with reactants, and one-third with void volume.

Common mistakes to be avoided when operating ball mills are as follows.

• The use of too few or too many milling balls, *e.g.*, the erroneous use of only one 10 mm milling ball inside a 50 mL vessel.

• The use of milling balls with the wrong size, *e.g.*, the use of overly large (>20 mm) or small (<5 mm) milling balls in vibrational ball mills.

• The use of incompatible balls and vessels, *e.g.*, zirconium oxide balls in steel vessels.

• Underestimation of pressure build-up during the reaction, for example, risk of explosion during work with permanganates, carbides, *etc.*

Unlike ball milling, reactive extrusion facilitates continuous and scalable synthesis.^92^ An extruder comprises a feed port, one (single-screw extruder; SSE) or two (twin screw extruder; TSE) screws placed inside a heatable barrel, and an exit port.^93^ In an SSE, the screw diameter gradually increases in the direction from the feed port towards the exit port, which increases the compressive force acting on the material as it passes the barrel. The two screws of a TSE can rotate in the same or opposite directions, applying shear to the sample.^94^ Additionally, several different setups can be realised by exchanging screw segments for reverse segments, toothed segments, or kneading blocks to allow different mechanical actions.^92^ In polymer manufacturing, SSEs and TSEs are widely used for the mixing of molten polymers and additives.

## Mechanochemical reaction parameters

While classical synthetic procedures are mainly dependent on the nature and content (concentration) of the employed solvent, these parameters play a subordinate role in mechanochemistry. Although standard reaction parameters such as stoichiometry, temperature, and reaction time also apply, there are other important parameters that allow the sophisticated design of mechanochemical processes. For mechanochemical synthesis inside a high-speed ball mill, the nature of the milling material is a crucial variable along with the type of the ball mill itself.^95^ The density of the milling material is directly related to its moment of inertia, and therefore, the kinetic energy transferred to the sample.^22^ The most frequently utilised milling materials are agate (2.7 g cm^−3^), silicon nitride (3.3 g cm^−3^), corundum (3.8 g cm^−3^), zirconium dioxide (5.7 g cm^−3^), stainless steel (7.8 g cm^−3^) and tungsten carbide (14.3 g cm^−3^).^43,96^ Although heavier milling materials afford increased yields in several cases,^97,98^ this behaviour is not always directly transferable to mechanochemical polymer synthesis. The use of higher-density milling materials might not only enhance polymerisation, but also favour the destruction of the synthesised polymers.^20,53,99,100^ In addition to material density, chemical resistance also plays a significant role; hence, tungsten carbide and zirconium dioxide are primarily selected for lab-scale processing. For example, stainless steel corrodes upon contact with strong acids, which can lead to the contamination and rapid degradation of the milling material.^26^ Nevertheless, as it is significantly cheaper, stainless steel is the material of choice for large-scale industrial applications.

The size and number of milling balls are just as important as the nature of the milling material. For lab-scale applications, milling ball size usually ranges between 0.1 and 30 mm.^43^ To adjust the milling ball size, one has to keep the overall mass constant owing to the coherence between mass and energy.^101^ Furthermore, one can also mention the ball-to-powder mass ratio, which is defined as the total mass of milling balls divided by the total mill charge mass and can be adapted for process fine-tuning.^26^ In this regard, to ensure the adequate trajectories of milling media, one should maintain a uniform filling degree, which is ideally one-third of the grinding bowl volume.^69^ To achieve this optimal filling degree, one can change the reaction scale or the jar size or add a grinding auxiliary, often called a bulking agent,^3,95^ which should be chemically inert towards the reaction mixture.^74^ In terms of polymer mechanochemistry, one should ensure that the bulking agent can be removed from the usually solid product. For example, one can use salts (*e.g.*, NaCl) that are easily removable by water, which is beneficial in ecological terms.^102^ In contrast, silica is inappropriate for insoluble polymers, as it has to be removed with hydrofluoric acid, which may destroy the synthesised polymer. In particular, when one or more liquid starting materials are used, a bulking agent is of high importance, as it can function as an adsorbent and therefore allows one to circumvent the formation of sticky mud-like reaction mixtures, which hinders sufficient energy transfer.^26^

In a neat milling environment, only solids are involved, which creates a homogeneous sample distribution without the possibility of lump formation. In many cases, however, catalytic amounts of liquids can benefit mechanochemical syntheses.^25,103–105^ This liquid-assisted grinding (LAG) developed from the poorly quantified addition of a certain amount of solvent droplets to a mechanochemical reaction and was formerly referred to as solvent-drop or solvent-assisted grinding.^3,23,106,107^ Recently, the methodology has been defined by the LAG parameter *η*, which is the ratio between the amount of liquid (μL) and the total mass of solid components (mg) and usually lies between 0 (no liquid) and 1 (slurry or even homogenous solution).^3^ LAG can significantly enhance the yield or speed of a reaction, even if the starting materials are poorly soluble in the applied liquid.^108^ Although the latter phenomenon is scarcely understood, it is assumed that a small amount of liquid is permanently saturated with reactants or that a mobile surface layer is formed.^3,109^ Furthermore, larger amounts of liquids can act as porogenes and stabilise pore formation during the synthesis of porous polymers.^53,89^ For example, for the mechanochemical synthesis of poly(lactic acid) (PLA), LAG limited polymer chain degradation while speeding up propagation to afford a high-molecular-weight product.^99^ In addition, polymers can also be used as grinding auxiliaries (polymer-assisted grinding) during mechanochemical processing.^110–112^

Another very important parameter is the frequency (for vibrational mills) or rotation rate (for planetary mills) of ball milling.^113,114^ In either case, higher agitation frequency accelerates the movement of milling balls and thus increases their kinetic energy.^24,115^ Although the milling frequency is easy to adjust, the accompanying temperature change is difficult to regulate and must be taken into consideration (particularly considering the exothermal character of most polymerizations to compensate a negative entropy). In the context of polymer mechanochemistry, we again notice that a higher frequency might also result in the destruction of the synthesised polymers,^116,117^ as has been observed for different reaction systems and for both rotational and vibrational ball mills. This usually leads to the trend, that the chain length of a given polymer is growing with increasing reaction time for the first minutes of a reaction and consequently either plateaus if the longer polymer chains are degraded, or even decreases if the speed of degradation exceeds the reaction rate.^19^

This, however, is not surprising, as the degradation of polymers under mechanical force is systematically studied since the middle of the last century. Especially the detailed studies by Kausch on polymer degradation kinetics enabled novel insights into the mechanochemical degradation of polymers and led to the establishment of a variety of different systematic reports nowadays.^118–124^ Choi and Peterson have initiated systematic studies on mechanical force-induced polymer degradation by ball milling,^117^ revealing that milling frequency has the greatest impact on the degradation rate of polystyrene. For future studies, the balance between reaction and degradation rates must be considered to produce high-molecular-weight polymers.

## Mechanochemical synthesis of linear polymers

The solvent-free mechanochemical synthesis of polymers was pioneered by the groups of Kargin, Oprea, and Kuzuya,^19,125,126^ who investigated the polymerisation of vinyl monomers and showed that mechanical action can indeed deliver sufficient energy and mixing efficiency for polymer synthesis. Further research broadened the scope of mechanochemical polymer synthesis and revealed many features distinguishing it from conventional techniques.^20,90,127–129^ Unfortunately, the knowledge gained from studies of conventional solution-based systems is not always valid for solid-state mechanochemical polymerisation, as in the latter case, concurrent chain degradation, the heterogeneous nature of solid-state conditions, and unexpected reaction pathways result in previously unseen architectures.^20,99,125,130,131^ This chapter focuses on mechanochemical (chain-growth and step-growth) polymerisations affording linear polymers as well as on post-polymerisation modification and provides a summary of the current issues.

### Chain-growth polymerisation

#### Radical polymerization

During chain-growth polymerisation, unsaturated monomers are added to the active site of a growing polymer chain one at a time. Therein, the polymerisation of vinyl monomers constitutes the largest portion in polymer industry. While thermal activation is predominantly used in industry, other types of energy supply including photo-, electro-, and mechanical activations have also been investigated, each showing unique features and product structures as well as a distinctive monomer scope.^132–137^

Before discussing the essential aspects and details of mechanochemical chain-growth polymerisation, we outline a brief history of its development.

**Scheme 1:**
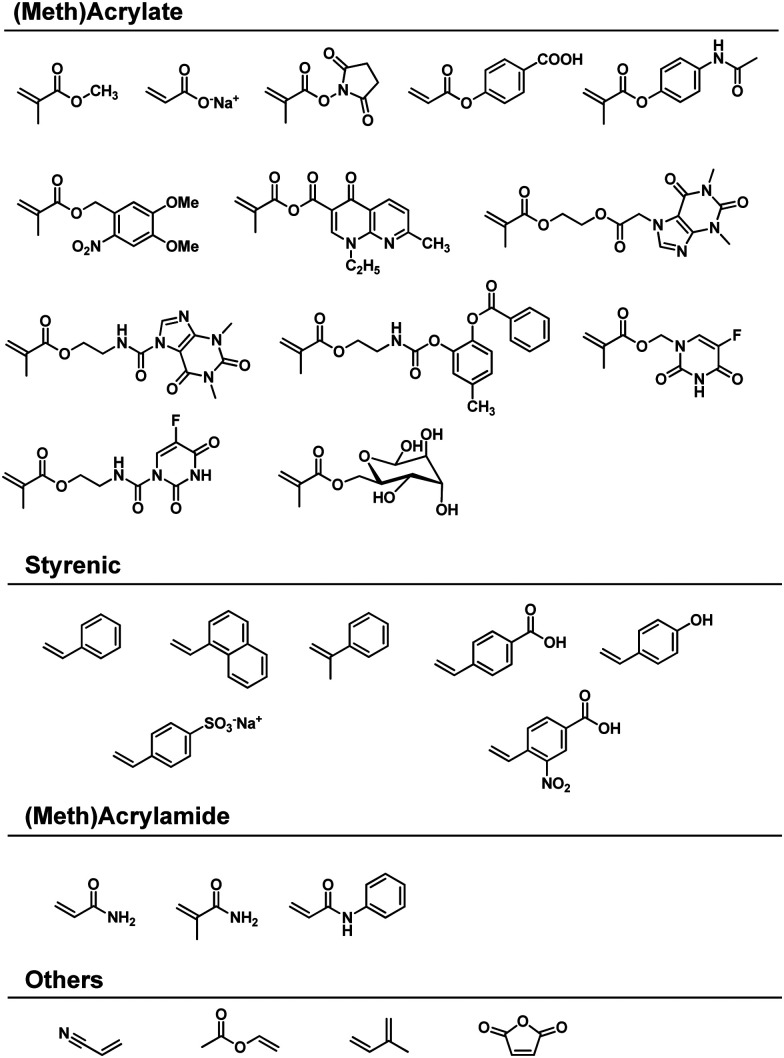
Reactive vinyl monomers used for solvent-free mechanochemical polymerisations.

In 1959 Kargin and coworkers disclosed the first mechanochemical polymerisation of methyl methacrylate and styrene in a vibrational ball mill.^17^ Shortly thereafter, the same group expanded the reaction scope to crystalline sodium acrylate and acrylamide.^125,138^ Continuing studies by Oprea in the 1980s and by Kuzuya in the 1990s covered many of the vinyl monomers known to undergo polymerisation (Scheme 1).^18^ The adopted strategy was used to convert (meth)acrylates,^19,130,139–145^ (meth)acrylamides,^125,126,141,146^ styrenes,^128,141,147–150^ and other structurally diverse vinyl monomers^149–152^ into the corresponding polymers under solvent-free ball-milling conditions. Moreover, the copolymerisation of these monomers has also been investigated,^125,130,140,149,153–155^ as is discussed below.

Conventional chain-growth polymerisation is usually initiated using compounds such as azobisisobutyronitrile (AIBN), benzoyl peroxide, or alkyl lithiums. However, mechanochemical ball milling can produce polymers from the corresponding vinyl compounds without any of these initiators, which makes the origin of initiation a topic of considerable debate.

Kargin and coworkers reported the mechanochemical polymerisation of MMA and styrene and claimed the importance of using NaCl and SiO_2_ as additives. According to them, the surface activation of these inorganic solids due to mechanical forces forms active radical or ionic sites, consequently causing initiation.^17,139^ This hypothesis was supported by several studies on active radicals and ionic sites formed by mechanical forces acting on the surface of inorganic solids.^156,157^

Later, Hasegawa continued related works using alumina, clay minerals, quartz, marble, and limestone activators. However, no direct observation of the propagating species was made.^158–164^

**Fig. 2:**
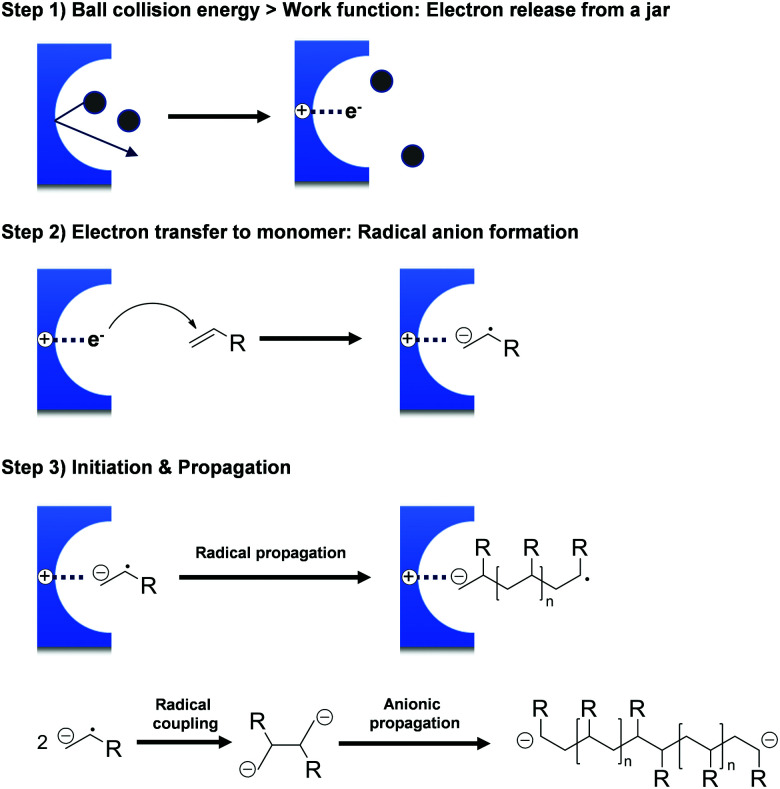
Mechanism of initiator-free mechanochemical polymerisation.

The hypothesis of ionic site formation at the surface of inorganic solid additives is not necessarily wrong, but its necessity is debatable, *e.g.*, the Oprea group reported that the ball-milling of acrylonitrile produces polyacrylonitrile even without additives.^151,165^ All monomers presented in Scheme 1 do not require external initiators for ball-milling polymerisation. Both Oprea and Kuzuya proposed that the initiation step involves electron transfer from the milling material to the substrate.^18,19,126,141^ Strong collisions between ball and vessel supply energy greater than the milling materials' work function, *i.e.* the threshold energy for electron emission. Electron transfer from the milling material generates a radical anion that triggers the observed polymerisation (Fig. 2). In agreement with this hypothesis, the addition of radical inhibitors such as oxygen, phenol, and hydroquinone slows down the reaction progress.^126^ Later, Kuzuya's group observed the propagating radical species using electron spin resonance (ESR) spectroscopy^146^ and reasoned that the milling jar material is critical for polymerisation.^19^

Highly efficient initiation was observed in a stainless-steel reactor, whereas no polymerisation was observed in a Teflon jar. Indeed, metal-based containers might more efficiently deliver electrons and thus facilitate initiation; however, monomer self-initiation cannot be excluded completely.^166,167^

**Fig. 3:**
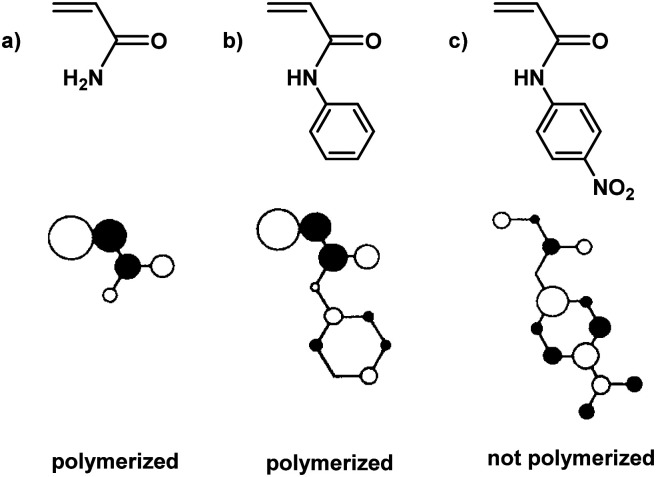
LUMO localisation of selected acrylamide monomers.

To elucidate this topic further and understand why some monomers do not show any reactivity during ball milling, Kuzuya and coworkers conducted theoretical studies on the relationship between monomer structure and reactivity.^19,132^ They found that the structure of the lowest unoccupied molecular orbital (LUMO) of the monomer was directly correlated to initiation efficiency (Fig. 3). The shape of the LUMO was further proposed to be a more pertinent factor than its energy. Only monomers with LUMOs localised on the vinyl unit underwent solid-state polymerisation (Fig. 3a and b). When the LUMO extended to substituent orbitals, radical anion formation was sluggish (Fig. 3c).

**Fig. 4:**
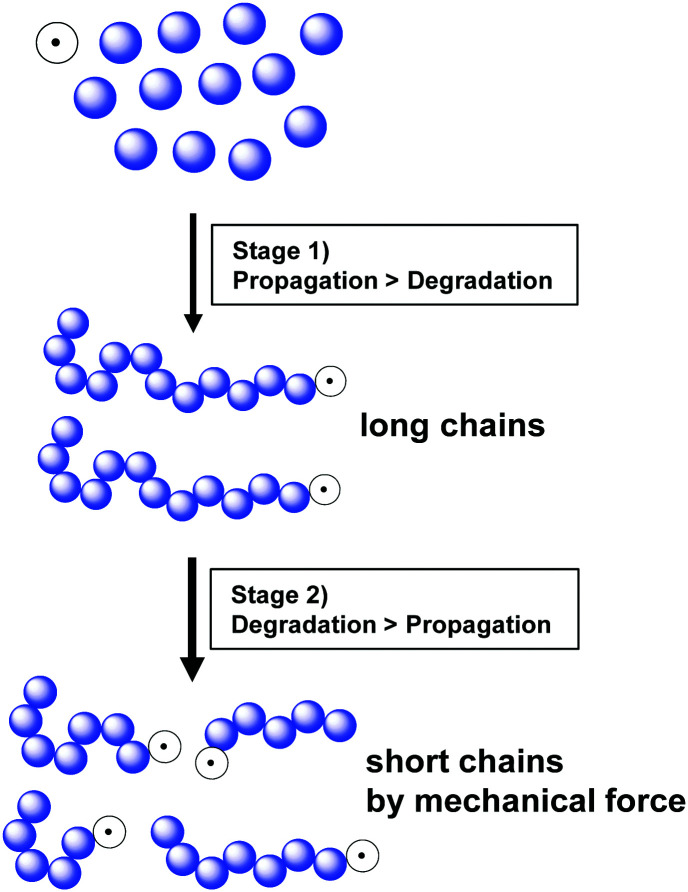
Schematic of mechanochemical polymerisation and subsequent chain degradation.

Kuzuya and coworkers further monitored conversion, radical concentration, and molecular weight changes during the reaction of a methyl acrylate monomer.^19,146^ At the beginning (<5 min), only a small amount of the active radical species was generated. High monomer concentration resulted in fast chain propagation, and chains with the highest molecular weight were produced at a monomer conversion of 10%. In continuation of the reaction a fast monomer consumption and a decrease in molecular weight was observed. This is because after reaching the threshold molecular weight, the polymer chains undergo degradation by ball milling and the homolytic bond dissociation produces short polymers with an active radical chain-end (macroradical) resulting in an increase in radical concentration. This phenomenon had two implementations, *i.e.*, fast polymerisation within 5–20 min and a maximum molecular weight limited by chain degradation. After 20 min, the polymer chains experienced degradation until the minimal molecular weight was reached. Fig. 4 summarises this reaction mechanism, showing that in the early state, propagation is dominant (stage 1) and once high-molecular-weight chains appear, mechanical force-induced chain degradation becomes dominant (stage 2), which results in molecular weight reduction.

In their studies, Kuzuya and coworkers obtained mechanochemically prepared polymers with narrow molecular weight distributions, ascribing this to acute polymerisation induced by mechanical forces.^145,168–170^ However, it is important to understand that this behaviour was mainly caused by mechanical degradation and not by controlled polymerisation kinetics. Hence, full control over chain length, as in the cases of living or controlled polymerisation, has scarcely been realised mechanochemically.

Today, chain length can be adjusted using reversible deactivation radical polymerisation.^171,172^ Although mechanochemical radical polymerisation has been studied for over 60 years, the related controlled polymerisation has not been realised until Cho and Bielawski's recent report.^128^ These researchers developed a mechanochemical atom radical transfer polymerisation (ATRP) of solid 2-vinylnaphthalene. The polymerisation system comprised Cu powder, CuBr, tris(2-pyridylmethyl)amine, and 1-bromo-1-phenylethane and allowed for high monomer conversion, linear chain growth proportional to the monomer consumption rate, and narrow dispersity. The realisation of control over chain length using different monomer-to-initiator ratios confirmed the reversible activation/deactivation mechanism previously observed for the solution state. The retardation of polymerisation in the presence of TEMPO and the isolation of phenylethyl-TEMPO also supported a radical mechanism. Despite this great success, it needs to be emphasised that chain degradation still remained uncontrolled, limiting the polymer molecular weight (*M*_n_).

**Fig. 5:**
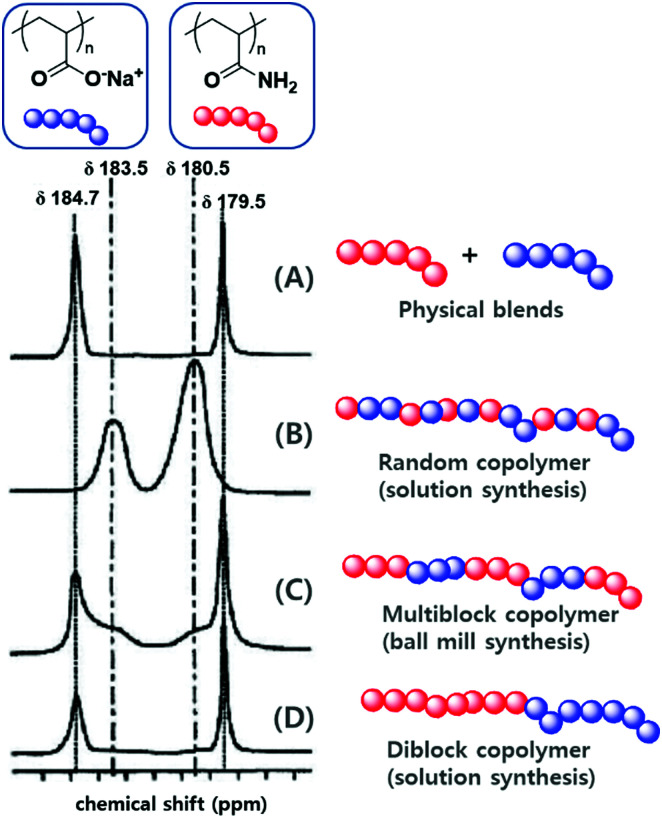
^13^C NMR signals of the carbonyl groups of (A) a physical blend of polyacrylamide and poly(sodium acrylate), (B) a random copolymer of polyacrylamide and poly(sodium acrylate) prepared by solution-phase radical polymerisation, (C) a multiblock copolymer of polyacrylamide and poly(sodium acrylate) prepared by ball milling, (D) a diblock copolymer of polyacrylamide and poly(sodium acrylate) prepared by ball milling. Reproduced with permission from ref. 130. Copyright (2000). The Pharmaceutical Society of Japan.

The differences between mechanochemical and solution-phase radical polymerisation are particularly obvious for copolymerisations.^125,130^ As an illustrative example, Fig. 5 shows the ^13^C NMR spectra of four different polymers based on polyacrylamide and poly(sodium acrylate), namely (A) a physical blend, (B) a copolymer prepared by solution-phase radical polymerisation, (C) a copolymer prepared by mechanochemical solid-state polymerisation, and (D) a diblock copolymer.^130^ The carbonyl environments of (A) and (D) are similar because of the lack of an acrylamide–sodium acrylate sequence in the polymer chains. The spectra of products obtained by solution-phase (B) and solid-state (C) polymerisation, however, showed signals at *δ* = 183.5 and 180.5 ppm, which corresponded to heterojunctions. Whereas the completely random copolymer (B) exclusively showed heterojunction peaks, polymer (C) showed the signals of both homojunctions (major) and heterojunctions (minor). Consequently, the formation of a multiblock-type polymer is anticipated if a mechanochemical pathway is involved. These results support those of the early study of Kargin and coworkers on the copolymerisation of acrylamide and methyl methacrylate (Table 1).^125^

**Table 1:** Copolymerisation of acrylamide (AA) and methyl methacrylate (MMA) at various temperatures

Monomers		Temp. (°C)	Condition	Relative reactivity ratios	
M1	M2			*γ*_1_	*γ*_2_
AA	MMA	70	Benzene, 0.15% AIBN	4.2	0.12
		−65	No solvent, 1% NaCl	36.6	4.2

Under the conditions of conventional solution polymerisation (70 °C in benzene, AIBN), the addition of acrylamide was dominant regardless of the active chain-end structure because of the higher reactivity of acrylamide compared to that of methyl methacrylate. In the initial stage, polyacrylamide was primarily produced. However, solid-state polymerisation at −65 °C afforded a strikingly different product. In this case, the heterogeneous distribution of solid monomers caused each monomer particle to become a domain in which one monomer exists excessively. Thus, the concentration factor overrode end-group reactivity, especially the heteropropagation tendency of methyl methacrylate end radicals. Each chain end preferentially underwent homopropagation before entering another monomer particle, which afforded a multiblock-like copolymer structure. In addition, the copolymerization of orthogonally soluble monomers, *i.e.*, when monomers lack solubility in a common solvent, can profit from a mechanochemical pathway. Bielawski and Cho presented the copolymerization of hydrophobic 2-vinyl naphthalene and ionic sodium styrene sulfonate in their above mentioned ATRP study.^128^

The mechanical fracture of polymers and the resulting physical changes have been known for a long time, and the underlying mechanism has been of interest since the dawn of polymer science.^7–9^ In 1952, Watson and coworkers showed that mechanically cleaved rubber produced polymer fragments with a radical end, known as macroradical.^173,174^ That these species indeed initiate polymerization reactions was then reported by Goto and Fujiwara in 1963.^175^

**Fig. 6:**
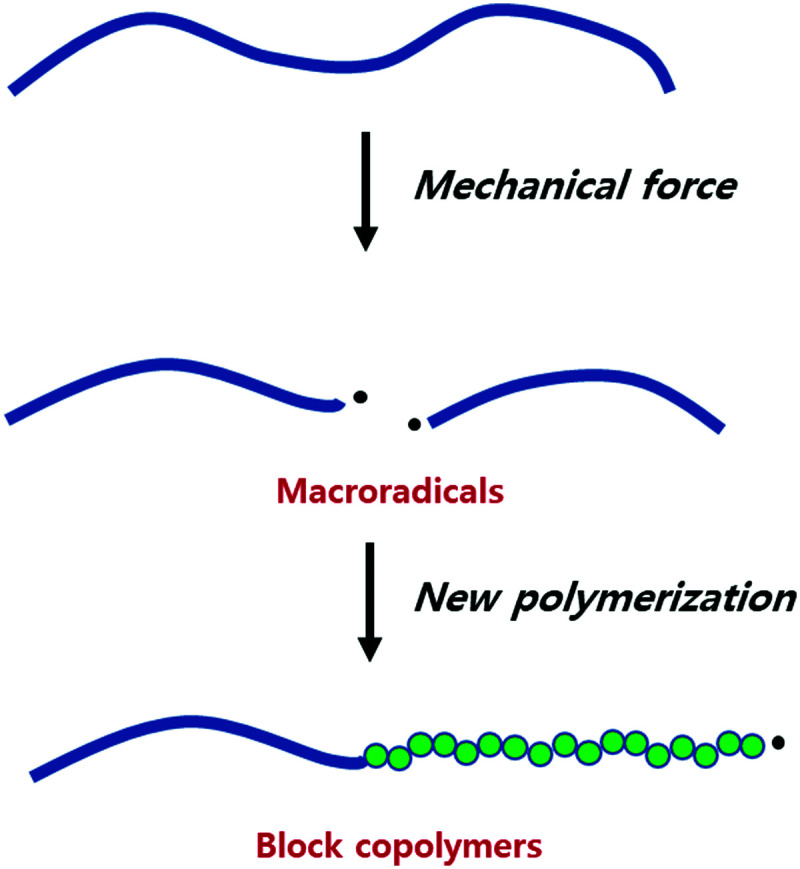
Illustration of macroradical formation-induced block copolymerisation.

The species obtained by the mechanical breakdown of poly(vinyl acetate) (PVA) in a solution of vinyl acetate (VA) were found to consume VA. The fact that this consumption was quenched in the presence of hydroquinone supported radical propagation. This experiment has been extended to the synthesis of various block copolymers, especially those not accessible by conventional methods (Fig. 6).

The involvement of mechanically produced macroradicals was also reported by Sohma and coworkers in their study on the mechanochemical synthesis of polytetrafluoroethylene (PTFE) block copolymers.^131^ ESR spectroscopy showed that the mechanical fracture of solid PTFE produced PTFE macroradicals, which, in view of their low mobility, were not terminated under anaerobic conditions and initiated chain propagation upon the introduction of monomers such as MMA, VA, and ethylene. This method allowed the synthesis of block copolymers (*e.g.*, PTFE-*b*-PMMA, PTFE-*b*-PVA, and PTFE-*b*-PE) difficult to obtain under common polymerisation conditions. Kuzuya and coworkers also used the macroradical formation and concurrent polymerisation of solid-state monomers to produce amphiphilic polymers. Specifically, the ball milling of polymethacrylate,^142,144^ poly(4-vinylpyridine),^170,176^ or cellulose^168^ produced the corresponding macroradicals that initiated the radical polymerisation of solid-state monomers to afford block copolymers of various compositions.

The capping of active macroradicals with functional small molecules is another way to obtain end-functionalised polymers. Ito's group realised this concept through ball milling.^177^ Polymeric macroradicals formed *in situ* under ball milling conditions underwent radical coupling reaction with a prefluorescent nitroxide based reagent. A series of luminescent polymeric materials were obtained from polystyrene, polyethylene, poly(methyl methacrylate), poly(phenylene sulfide), and polysulfone, showing off its synthetic versatility.

#### Ring-opening polymerisation (ROP)

Ring-opening polymerization (ROP) is a type of chain-growth polymerization, in which a reactive chain end attacks and opens cyclic molecules to form a long polymer. It is an especially popular method for the synthesis of medical polymers such as poly(lactic acid).^178–180^ Although ROP is typically carried out in solution or at high temperatures, the team led by Kim investigated the solvent-free mechanochemical polymerisation of lactide and trimethylene carbonate induced by ball milling. These studies are particularly important as for the first time the mechanochemical perspective, *i.e.* milling parameters such as milling material, ball size, the energy impact, was put on polymerization reactions.^91,99,181^ Recently, Lamaty and Giani expanded the scope to the ROP of morpholine-2,5-dione using ball milling.^182^

Initially, the Kim group showed that conventional organic superbase catalysts such as 1,8-diazabicyclo(5.4.0)undec-7-ene (DBU) and triazabicyclodecene can polymerise solid lactide and trimethylene carbonate without a solvent and that ball milling enables efficient initiator-catalyst mixing even at very low concentrations. The number and mass of the milling balls significantly influenced mechanochemical conversion.^99^ At a constant total mass of moving parts, a comparison of one 12 mm ball with five 7 mm balls showed that faster conversion was achieved in the latter case (89% *vs.* 49%, respectively). A similar trend was observed for poly(trimethylene carbonate) synthesis,^91^ and it was concluded that the magnitude of the impact energy (single event) was more influential than the total energy input.

**Fig. 7:**
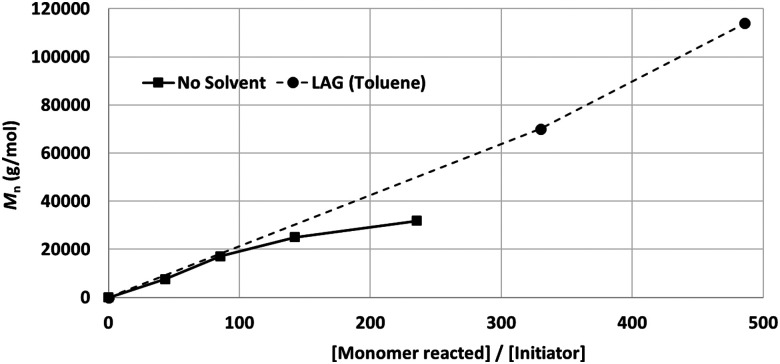
Effect of LAG on the production of high-molecular weight PLA.

While neat grinding resulted in the anticipated chain degradation after a DP of 100 (Fig. 7, solid line), LAG selectively retarded chain degradation and enhanced the polymerisation rate (Fig. 7, dotted line).^99^ Among the tested liquid additives, toluene (*η* = 0.2 mL mg^−1^) exhibited the best effect, *i.e.*, no signature of chain degradation was observed up to 1 × 10^5^ Da. The exact mechanism of LAG-mediated chain protection is not clear, particularly considering that this effect was not observed for poly(trimethylene carbonate) synthesis. We hypothesise that the protective effect of LAG is due to the combined influence of the absorption of impact energy, material lubrication, and mass transport enhancement.

Mechanochemical ROP can be a powerful technique for obtaining block copolymers.^181^ For instance, macroinitiators with terminal hydroxy groups, such as poly(ethylene oxide) (PEO), can be extended by lactide polymerisation. As PEO is hydrophilic and is not miscible with lactide and the organic catalyst (DBU), initial trials using high-molecular-weight PEO were unsuccessful because of poor mixing. This problem was overcome by increasing milling energy through the use of larger milling balls and LAG (THF, *η* = 0.2 mL mg^−1^), which resulted in the successful synthesis of triblock copolymers.

A warrantable concern for mechanochemical polymerisation reactions is whether they indeed proceed in the solid state. Therefore, the authors of the latter study performed instant temperature measurements at the end of polymerisation, revealing only a slight increase to 44 °C, which is below the melting temperature of PLA (110 °C), PEO (60 °C), and lactide (95 °C). As the resulting mixture was a fine powder, the formation of a eutectic mixture occasionally seen in mechanochemical synthesis was ruled out.

### Step-growth polymerization

Step-growth polymerisation refers to the successive reaction of multifunctional monomers to afford dimers, trimers, oligomers, and long-chain polymers. According to Carothers equation, the formation of the desired polymer chain is warranted by high reaction efficiency and low side-reaction probability.^183^ The fact that mechanochemical reactions can proceed at maximal monomer concentration (*i.e.*, in the absence of a solvent) results in increased reaction rates and efficiencies, which makes mechanochemical techniques particularly suitable for step-growth polymerisation. At the same time, the lack of the solubility limitation allows the preparation of polymers hardly accessible in solution, such as unsubstituted polyphenylenes.

**Fig. 8:**
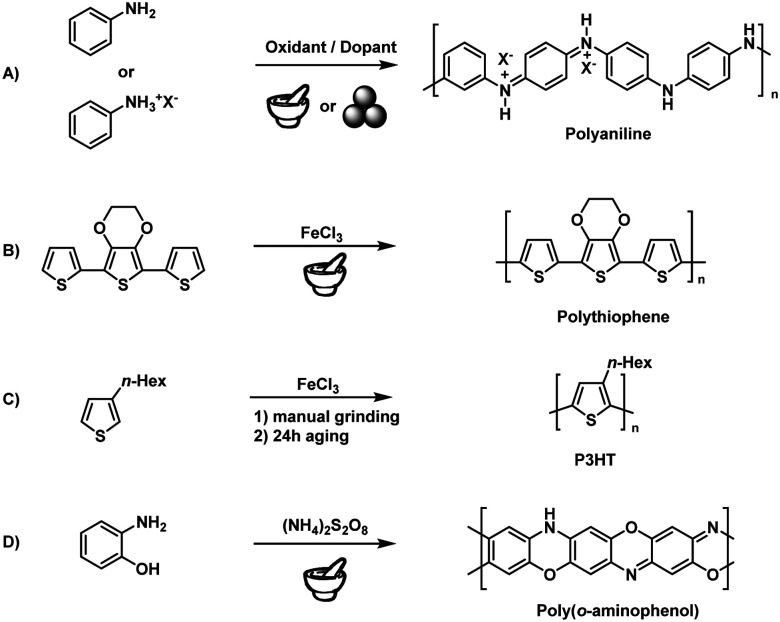
Solid-state conducting polymer synthesis: early examples.

Early attempts relied on the simple manual grinding of reactive monomers using mortar and pestle. In 2002, Qu and coworkers described the solid-state synthesis of doped polyaniline (PANI)^184–187^ based on the 30 min hand-grinding of frozen aniline at −20 °C with the inorganic acid H_4_SiW_12_O_40_ (Fig. 8A). This technique has drawn much attention in the polyaniline community because of its synthetic simplicity, greenness, and unique properties but exhibited all the above mentioned drawbacks of manual grinding concerning reproducibility and scalability.^188,189^ To meet these challenges, Kaner and coworkers applied ball milling to the mechanochemical synthesis of PANI.^190,191^ The authors used anilinium salts such as anilinium chloride and oxidants such as ammonium peroxysulphate, studying the effect of dopants and other derivatised anilines. Posudievsky and coworkers showed that the molecular weight of PANI prepared by ball milling was similar to that of PANI prepared using a solution-based method. Here, one should note that PANI was produced by a facile polycondensation reaction that does not require high mechanical energy. For this reason, syntheses could be performed even with simple mortar-and-pestle grinding. An in-depth study of the influence of grinding parameters was, unfortunately, not considered necessary at that time.

Solid-state syntheses of other conducting polymers have also been reported. Nurulla and coworkers synthesised poly(thiophene) derivatives by the hand-grinding of terthiophene and FeCl_3_ (Fig. 8B), while Takahara and coworkers used 3-hexylthiophene to synthesise poly(3-hexylthiophene) (P3HT) by grinding and subsequent aging for 24 h (Fig. 8C).^192–194^ Notably, aging seemed to be an important factor. Whereas room-temperature aging only yielded low-molecular-weight products (*M*_n_ = 3000 Da), Kumar's group showed that aging at elevated temperatures (100 °C) produces high-molecular-weight polymers in good yields, using poly(3,4-propylenedioxythiophene) as an example.^195^ Zoromba synthesised poly(*o*-aminophenol) using an automated mortar-and-pestle grinder (Retsch RM200) (Fig. 8D).^196^

Similar to chain-growth polymerisation, in the 2010s, mechanochemical step-growth polymerisations advanced their focus from a purely chemical perspective to a more mechanochemical point of view that involves the study of milling parameters. In 2014, Swager and coworkers studied mechanochemically promoted solid-state Gilch polymerisation with an emphasis on these parameters.^20^ High-speed ball milling of bis(chloromethyl)benzene (monomer) and ^*t*^BuOK (base) produced poly(phenylene vinylene) (PPV) with a molecular weight of up to 40 000 Da in less than 1 h with high reproducibility, whereas the solution process often required more than 24 h and afforded a large deviation in molecular weights between each synthesis. Notably, no PPV was formed when slow vibration or small balls were used, which suggests that a certain mechanical energy threshold needs to be overcome for efficient synthesis. The progressing chain degradation with increasing reaction time limited the molecular weight to ∼40 kDa.

**Fig. 9:**
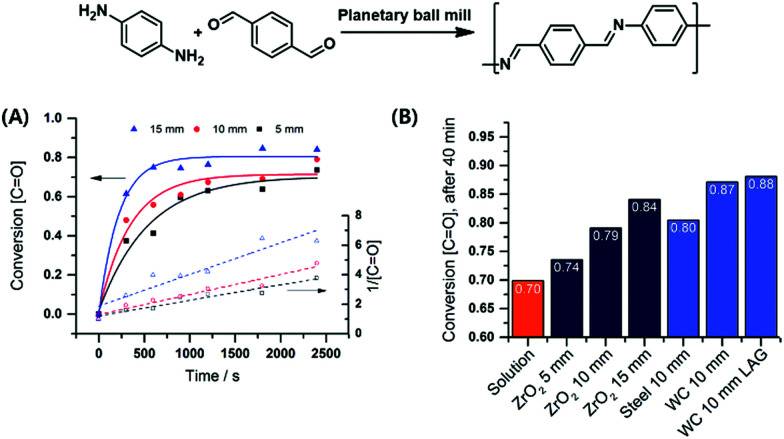
Polycondensation *via* highly efficient mechanochemical Schiff base formation: effects of (A) ball size and (B) milling materials. Reproduced with permission from ref. 197. Copyright (2016). The Royal Society of Chemistry.

Borchardt and coworkers continued this focus on the impact of milling parameters on step-growth polymerisation by investigating a series of mechanochemical polycondensations of poly(azomethine), polyphenylene, and polyimide.^90,197–199^ The first investigation probed Schiff base formation from *p*-benzenedicarbaldehyde and *p*-phenylenediamine (Fig. 9),^197^ revealing that polymerisation efficiency was proportional to the density of milling materials and ball size. This result again demonstrates that the introduced mechanical energy promotes polycondensation. Mechanically prepared poly(azomethine) exhibited a longer chain length than that prepared by solution-phase methods, which was ascribed to the fast precipitation of polymers during classical synthesis.

However, this trend holds only to a certain extent, as demonstrated by the same group for the Pd-catalysed Suzuki polycondensation of 1,4-dibromobenzene and 1,4-phenyldiboronic acid.^90,198^ In the presence of Pd(OAc)_2_ as the catalyst and K_2_CO_3_ as the base, 30 min milling in a planetary ball mill yielded *p*-polyphenylene (PPP) with a high DP of up to 164. Notably, heavy milling balls made from tungsten carbide afforded a lower DP than lighter milling balls such as those made from silicon nitride, as the higher impact energy in the former case resulted in chain degradation. This hypothesis was substantiated by post-polymer milling experiments in which the milling of freshly synthesised PPP decreased DP when the impact energy was sufficiently high. This type of mechanochemical polycondensation is comparatively robust to the change of substrate substitution pattern. In addition to the mentioned A2B2-type reaction affording linear polymers, AB-type homopolymerisations or A2B-type polymerisations affording hyperbranched polymers were also possible.

A major breakthrough in the field of step-growth polymerisation was the development of direct mechanocatalysis, in which case the reaction is catalysed by milling balls and not by intentionally added soluble or solid-state catalysts, as in the abovementioned polymerisations. Vogt *et al.* used Pd milling balls to promote the conversion of 4-bromophenylboronic acid to PPP within a ball mill,^198^ achieving high DPs of up to 199 in the absence of ligands or salts. This study substantiated the strong influence of impact energy and the need to adjust it by tuning milling parameters. Indeed, a certain critical impact energy is necessary, as milling at <200 rpm did not induce any conversion; however, excessively high impact energies led to chain degradation.

The impact energy can be adjusted by tuning the hardness of the milling vessel. If Pd milling balls are used in soft Teflon vessels and not in zirconia or steel vessels, chain degradation is hindered because of the decrease in the power of impact events (elastic *vs.* inelastic collision). Moreover, this change also results in significantly reduced abrasion.

**Fig. 10:**
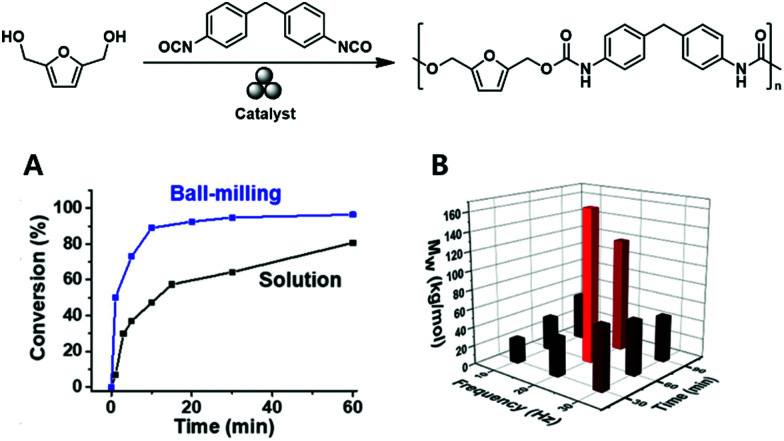
Mechanochemical solid-state polyurethane synthesis from a furan-diol: (A) milling optimisation and (B) comparison of ball-milling and solution-phase polymerisations in terms of reaction rate. Reproduced with permission from ref. 100. Copyright (2020). The American Chemical Society.

Song and coworkers made similar observations during their mechanochemical synthesis of polyurethane^100^ based on the vibrational ball milling-induced reaction of diisocyanates with a series of biomass-derived diols (*e.g.*, 2,5 bis(hydroxymethyl)furan) at ambient temperature (Fig. 10A). Whereas chain degradation occurred during fast (and hence, energetic) vibrational milling at 30 Hz, it was strongly hindered during slower milling at 20 Hz (Fig. 10B). Notably, the reaction was complete within 1 h, *i.e.*, was faster than the corresponding solution-phase reaction.

The synthesis of polyimides requires excess heating and long reaction times in solution synthesis. The ‘beat and heat’ process consists of ball milling of dianhydride and diamine, rapidly forming polyamic acid, and subsequent heating at 200 °C, shortening the process time to 1 h. Furthermore, the process enabled the synthesis of polymers inaccessible through solution-based processes.^199^

Such an economy of time was also observed by Shi and coworkers who synthesised polyamides from bis-benzoxazine and bis-isocyanate.^64^ The manual grinding of monomer mixtures in an agate mortar for 1 h produced a polyamide in good yield and with a high molecular weight comparable to that obtained in solution-phase reactions after 6 h.

### Post-polymerization modification

Post-polymerization modification (PPM), the chemical operation on an existing polymeric chain or side-functional group, provides new possibilities in polymer science, as it can be utilized to introduce chemical units that are not compatible with conventional polymerization methods.^200,201^ In addition, chemical modifications beginning from a parental polymer produce a library of functional polymers with identical chain-lengths and topologies, but with different functional groups, allowing the facile and acute study of structure–property relationships. However, a distinct disadvantage of post-polymerization modifications is the often limited solubility of the polymers, which narrows the opportunities for syntheses by conventional solvent-based methods. Recently, examples for solvent-free solid-state post-polymerization modifications have been reported, enabling to overcome the given limitation of solubility.

These procedures mainly focus on two different approaches, as it is either possible to change the functional groups of a polymer, or to modify the polymeric chain itself.

**Fig. 11:**
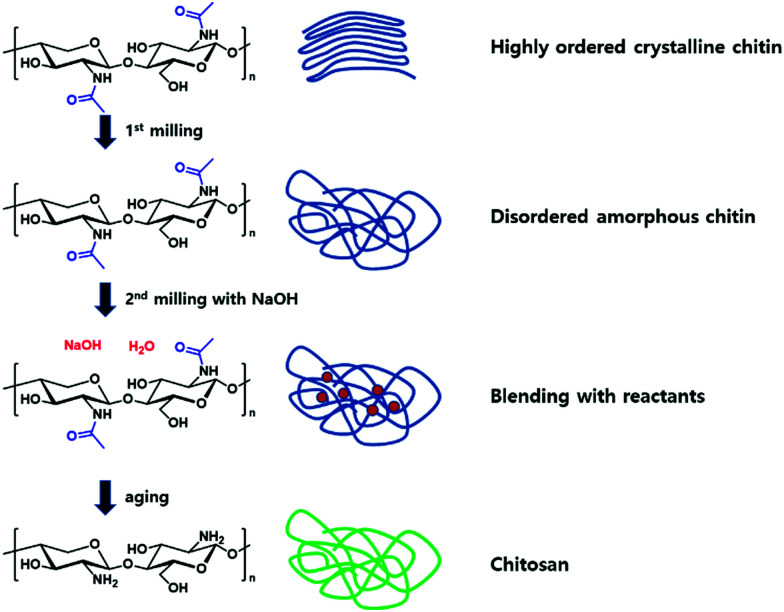
Schematic mechanochemical transformation of chitin to chitosan.

One distinct example for the latter was presented by the Moores group, reporting the transformation of chitin, the second most abundant biopolymer, into chitosan (Fig. 11).^202^ During the first step, ball milling of neat chitin softened the polymer to an amorphous disordered state, which enabled the mixing with NaOH during a second ball milling step. The mixture of chitin and the base underwent a final aging step under high humidity to facilitate deacetylation, giving high-molecular weight chitosan. Similar to aforementioned polymerization methods, the milling media density was found to be highly influential during the polymer chain modification. By the use of stainless steel as milling material, a higher deacetylation efficiency was exhibited, however, the viscosity decreased in comparison to the application of zirconia as milling material, attributed to the increase of chain degradations due to the higher energy impact of the heavier milling material.

**Fig. 12:**
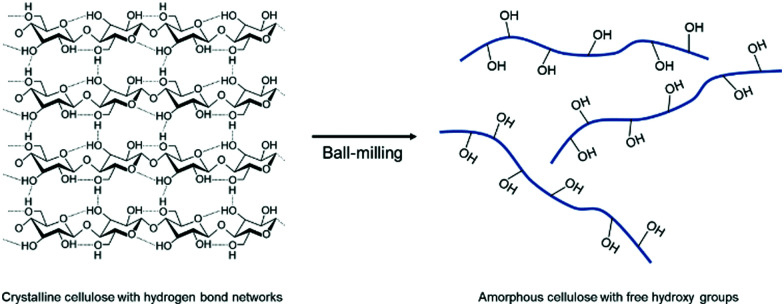
Phase transition and free hydroxy group generation by ball milling of crystalline cellulose.

The mechanochemical amorphization of well-known polymers was additionally examined by Hirotsu and coworkers by ball milling of crystalline cellulose.^203^ Since the strong hydrogen bonds between the cellulose chains limit the processability in solution, the group utilized planetary ball milling to turn crystalline cellulose into an amorphous state by breaking the hydrogen bond network between the hydroxy groups. As a result, the free hydroxy groups can participate in chemical reactions more efficiently (Fig. 12).

Based on this approach the modification of the polymers’ functional groups was possible in a further step. Moores and Friščić attempted the phosphorylation of milling induced amorphous cellulose by a mechanochemical functionalization with phosphorus pentoxide.^204^ To highlight the versatility of this mechanochemical post-polymerization modification, various polymers were brought to reaction with P_2_O_5_ by ball milling, including cellulose nanocrystals, poly(ethylene oxide), poly(vinyl alcohol), poly(vinyl chloride), and lignin. Although the approach enabled the circumvention of the conventional energy-intensive solution method, corrosive phosphoric pentoxide was needed to be applied for the mechanochemical process, which is a distinct drawback for a green synthesis procedure.

Another drawback of the mechanochemical modification of polymer functionalities is a lack of process control, as presented by Hirotsu and Qiu.^205,206^ By milling polypropylene, maleic anhydride, and initiator together, polypropylene macroradicals were generated. This was achieved by either direct mechanical chain degradation or by initiator-induced chain disproportionation, whereupon a reaction with maleic anhydride occurred, yielding a maleic anhydride grafted polypropylene (MAPP). Since the radical process is uncontrollable under ball milling conditions, the resulting functional polymer exhibited an undesirable high melt flow index, attributed to chain crosslinking reactions. Interestingly, the mechanochemically prepared MAPP showed a lower flow ability than those obtained in melt, thus less chain scission happened during ball milling.

**Fig. 13:**
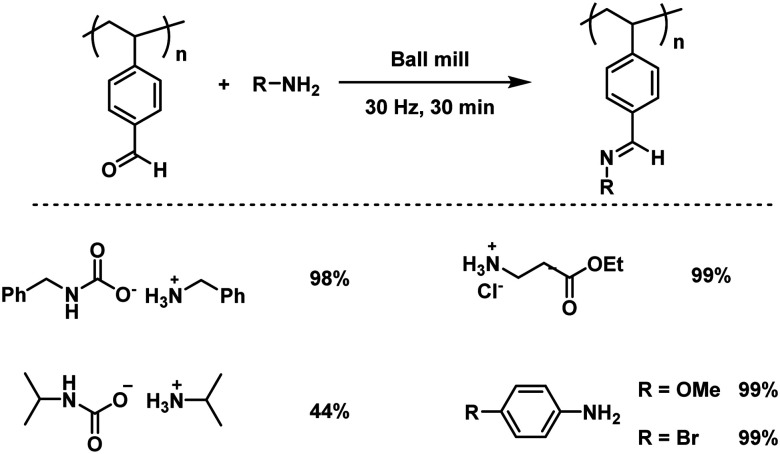
PPM of an aldehyde-polymer with amines induced by high-speed ball milling.

Since uncontrollable conditions are undesirable, the Kim group reported the first regulated mechanochemical post-polymerization modification of the polymer main chain functionalities.^127^ Thereby, a quick condensation reaction between a model aldehyde-polymer, poly(4-vinyl benzaldehyde), and assorted amines or solid amine surrogates was promoted by ball milling (Fig. 13), resulting in a complete aldehyde conversion towards the imine within 30 minutes. This functionality modification showed no sign of uncontrolled side reactions and the molecular weight of the polymer increased gradually, while the dispersity remained unchanged. Interestingly, the reaction with an ionic substrate, ammonium carbamate, did not hamper the reaction rate regardless of the immiscibility of the reactants. In a further approach Kim and Kim additionally verified that the immiscibility is indeed not a limiting factor for mechanochemical post-polymerization modifications, as they achieved the conjugation of hydrophobic molecules and an ionic polymer, poorly soluble in organic solvents, by ball milling.^207^

**Fig. 14:**
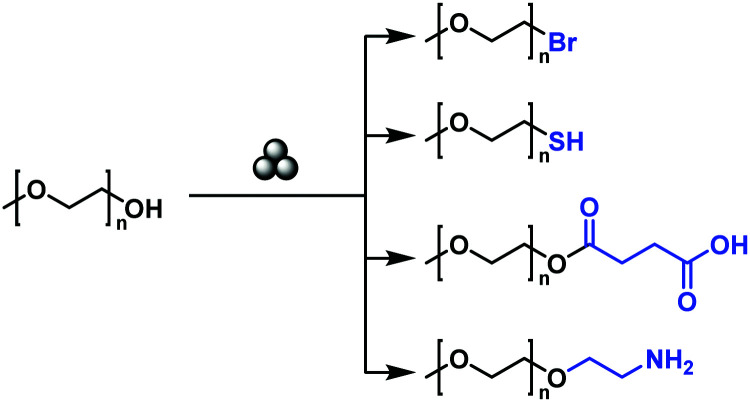
Mechanochemical ω-functionalisation of poly(ethylene glycol).

In addition to the modification of the main chain functionalities, it is furthermore possible to modify the chain end functionalities of a polymer by mechanochemistry, even in spite of a low-end group concentration. Moores and Friščić realized the solid-state mechanochemical ω-functionalization of poly(ethylene glycol), by the activation of the terminal hydroxy group with a base (Fig. 14).^208^ Thereby, a series of end-functionalized PEGs was obtained with moderate to good yields without any solvent. The report used low molecular weight PEG (*M*_n_ = 750 and 2000 kDa), in which no degradation was anticipated.

### Challenges and outlook

Carothers equation, describing the influence of monomer concentration on the polymerization efficiency, showcases why the solvent-free and thus highly concentrated environment of a ball mill is in principle well-suited for step growth polymerizations. Likewise, the possibility to form radicals during the mechanical impact event of milling showcases that this technique is also well-suited for initiator-free chain-growth polymerizations. Nevertheless, mechanochemical polymerization is still in its infancy and there are many aspects urging for improvement. For example, polymerization kinetics established for classical homogeneous systems are not comparable to what is observed in solid-state. The fast initiation over propagation is a criterion for narrow molar mass distribution, but the physical blending of initiator and monomer brings an inherent delay and unevenness.

Additionally, a tailored polymer synthesis is urgently necessary, as in many examples chain growth competes with chain degradation. This results in a dilemma between the utilization of high impact conditions for higher yields and faster reaction kinetics and of low impact conditions for suppressing chain degradation. The lack of understanding of the mechanism of chain degradation led to a trial and error-based research. In this context, the impact of LAG, temperature, and post-synthesis aging for instance is not understood so far.

Moreover, the scalability is a challenging issue for future commercialization. For industrial applications, continuous processes are preferred to enable the synthesis of kilograms or even tons per hour. However, the tool that was mostly presented herein, ball milling, generally could not meet this criterion. Therefore, the engineering of ball mills or the transfer to other mechanochemical methods, such as to extrusion, should be advanced further.

Another drawback is the reproducibility of mechanochemical reaction protocols, as it is often depending on the use of the same or similar equipment. Problems usually arise if rather exotic devices or home-built vessels are used. Lately, we have observed a trend that many mechanochemical laboratories use the same mills and thus this issue should soon be behind us.

Although, there are still distinct deficiencies of mechanochemical polymerization reactions that need to be circumvented in the future, the advantages of solvent-free synthesis protocols, such as the avoidance of toxic solvents and the circumvention of solubility-based limitations, render the whole area as highly important for future applications. We are certain that other common polymerization techniques, such as polyaddition reactions, will soon be transferred into solvent-free mechanochemical protocols. One interesting example would be the establishment of controlled/living polymerizations, which is highly required as it allows the block copolymer synthesis by sequential monomer addition.

Highlighting this, there is plenty of room for new reactions to be transformed into a mechanochemical process, but even more desirable is a deeper understanding of the underlying principles of mechanochemical polymerization. Moreover, it is highly necessary to monitor and follow mechanochemical polymerization reactions *in situ*. Modern *in situ* techniques based on Raman spectroscopy or XRD, which are coupled directly into the vibrating mill will help to elucidate this missing information in the future.

## Mechanochemical synthesis of porous polymers

Porous polymers have diverse applications in fields of industrial relevance^209,210^ such as catalysis,^211–214^ molecular separation,^215,216^ gas storage,^217–222^ and energy storage in batteries and supercapacitors.^223,224^ These materials feature large pore volumes and high specific surface areas and can be classified as macroporous (pore diameter > 50 nm), mesoporous (pore diameter = 2–50 nm), or microporous (pore diameter < 2 nm).^225,226^ Categorisation by material classes opens up a broad range of various polymer groups such as porous coordination polymers (PCPs)^227–229^ or porous organic polymers (POPs).^230,231^

While many classical solvent-based syntheses require expensive starting materials or catalysts,^232,233^ mechanochemistry obviates the need for solubilising groups and allows one to avoid low DPs due to the rapid precipitation of products.^234^ The need to reduce solvent waste has inspired several studies on the general mechanochemical synthesis of porous materials^235,236^ such as porous carbons,^237–248^ porous coordination polymers^249,250^ (*e.g.*, metal–organic frameworks (MOFs)^251–255^), and purely organic polymers.^53,102,256–260^

Up to now, porous polymers have been exclusively synthesised by step-growth polymerisation, while particularly using polycondensation reactions. Although this allows the following chapter to be integrated into the previous one, the available reports are much more numerous than in the case of linear polymers and often have a different focus because of the difference in the relevant material properties. For instance, the specific surface area, pore size, and pore volume are more important properties than DP or dispersity degree. Moreover, as long as the respective polymers are insoluble in common organic solvents, even a lower degree of polymerization might be favourable in order to ensure a better pore accessibility. Herein the focus is therefore shifted from the degree of polymerization to the specific surface areas of the porous polymers. As coordination polymers such as MOFs have been discussed in detail in previous reviews, we exclusively focus on porous polymers built from covalently bonded monomers and identify three major techniques to mechanochemically build up these polymers, namely Scholl polymerisation, Friedel–Crafts polymerisation, and imine/enamine/amide formation.

### Scholl polymerisation

The Scholl polymerization is a step-growth polymerization, in which aromatic compounds are coupled in the presence of (catalytic) amounts of a Lewis acid and an oxidant. Thereby, the *para* position of the respective monomers is favoured, firstly leading to the formation of smaller oligomers that react with each other to form an interconnected network. During this oxidative coupling reaction, the Lewis acid enables the elimination of hydrogen that is released as HCl. While this process takes long reaction times of up to 48 h and the necessity of often hazardous solvents in the classical solution based synthesis,^261–264^ mechanochemistry surpasses these drawbacks, rendering the approach extremely versatile to cross-link various monomers within very short reaction times of down to 5 minutes.

**Fig. 15:**
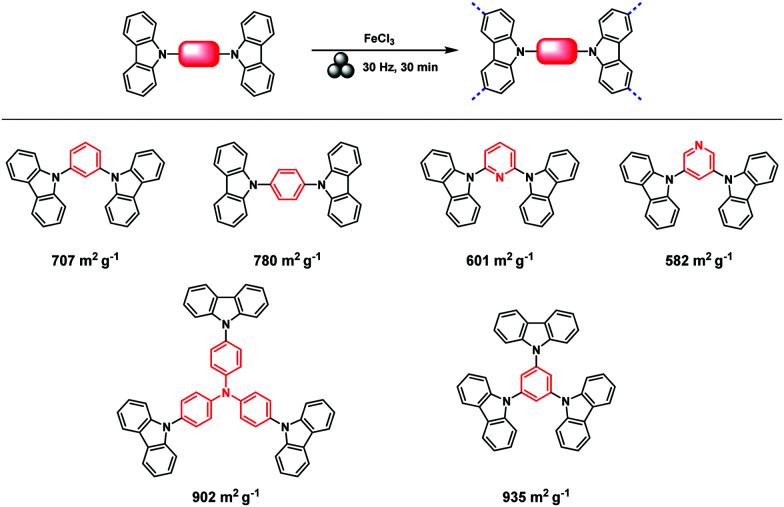
Top: Scholl model reaction for the generation of nanoporous conjugated polycarbazoles by ball milling. Bottom: Different building blocks and SSA_BET_ values of the corresponding polymers. Adapted with permission from ref. 256. Copyright (2017). The American Chemical Society.

**Fig. 16:**
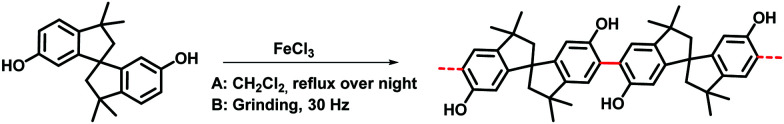
Classical (A) and mechanochemical (B) oxidative coupling polymerisation of 3,3,3′,3′-tetramethyl-2,2′,3,3′-tetrahydro-1,1′-spirobi[indene]-6,6′-diol. Reprinted with permission from ref. 265.

**Fig. 17:**
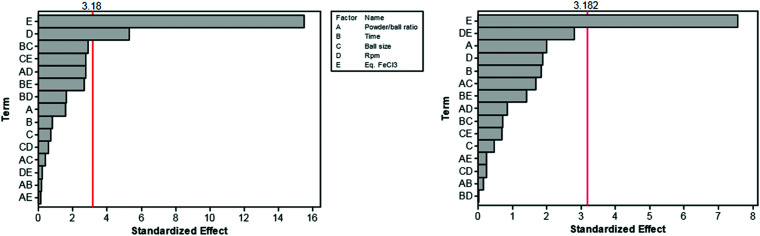
Design of experiment of the mechanochemical Scholl reaction yielding a microporous thiophene polymer. The most important influence onto the surface area (left) and the yield (right) are the eq. FeCl_3_ (E) and the frequency (D). Adapted from ref. 102 with permission from The Royal Society of Chemistry.

The first proposal in this regard was presented by the group of Dai, adapting the mechanochemical oxidative coupling procedure towards conjugated nanoporous polymers (CNPs).^256^ In the given case various carbazole-containing building blocks were polymerized in a vibrational ball mill within 30 min and the building block geometry had a direct effect onto the SSA_BET_ of the polymers (Fig. 15). The most important detail, however, is the utilisation of anhydrous FeCl_3_ instead of FeCl_3_·6H_2_O as both a Lewis acid and an oxidant, which significantly simplified the synthetic procedure. The number of FeCl_3_ equivalents strongly influenced the reaction outcome. For instance, the polymer yield could be increased from 54% to 94% by increasing the amount of Lewis acid from 1.25 eq. to 5 eq. A similar result was obtained when the same group advanced this protocol to soluble nanoporous networks,^265^ starting from a ladder-like monomer structure (3,3,3′,3′-tetramethyl-2,2′,3,3′-tetrahydro-1,1′-spirobi[indene]-6,6′-diol) (Fig. 16). The synthesis was carried out with 1–4 mass equivalents of FeCl_3_ in a vibrational ball mill, and the increase in FeCl_3_ quantity was accompanied by an increased yield and a decreased solubility of the generated polymers in common organic solvents. Independently, Borchardt and coworkers came to the same conclusion when working on mechanically produced microporous thiophene polymers (Fig. 17).^102^ Despite facilitating high yields, large FeCl_3_ loadings strongly affected the SSA_BET_ of the polymer, with specific surface areas of almost 1900 m^2^ g^−1^ achieved for high-speed ball milling with 12 eq. of FeCl_3_. This is particularly surprising, as the reference polymer prepared using solution-phase chemistry featured a significantly lower SSA_BET_. The authors hypothesised that the instant precipitation of the product from the reaction mixture, which occurs in solution but not during mechanochemical processing, is responsible for this significant difference.^266^ Subsequent publications point towards the same, *i.e.*, confirm the important effect of FeCl_3_ loading on the outcome of mechanochemical Scholl reactions. A more detailed study of reaction parameters using qualitative and quantitative tools such as the design of experiments (Fig. 17) helped to identify crucial factors of influence. The investigation of the relations and cross-relations of reaction parameters such as milling time, ball-to-powder ratio, ball size, and milling frequency has shown that only FeCl_3_ loading and milling frequency have a significant impact on the reaction. Specifically, increased milling frequency induced pore collapse to decrease SSA_BET_, which was verified in a subsequent study on the Scholl polymerisation of 1,3,5-triphenylbenzene by the same group.^53^ In this work, a 5 min reaction afforded a polymer with a surface area of >1000 m^2^ g^−1^ in >99% yield. The insufficiently high impact energy observed at low milling frequencies, short milling times, and small milling material densities or ball sizes results in poor yields and modest specific surface areas. Despite providing quantitative yields, the opposite parameter combination was found to be similarly undesirable, as excessive energy input devastated internal porosity. Although the origin of the profound impact of the oxidant remains unclear, two factors might play a role. First, FeCl_3_ is very hygroscopic, and as many experiments were conducted under ambient conditions, moisture was rapidly drawn from the air. If only a small excess of the oxidant is used in the reaction, this might lead to deactivation below the required stoichiometry or to a pseudo-LAG experiment with an excessively high *η*.

Second, FeCl_3_ is often simultaneously used as a bulking material and thus influences the rheology of the milling process.

**Fig. 18:**
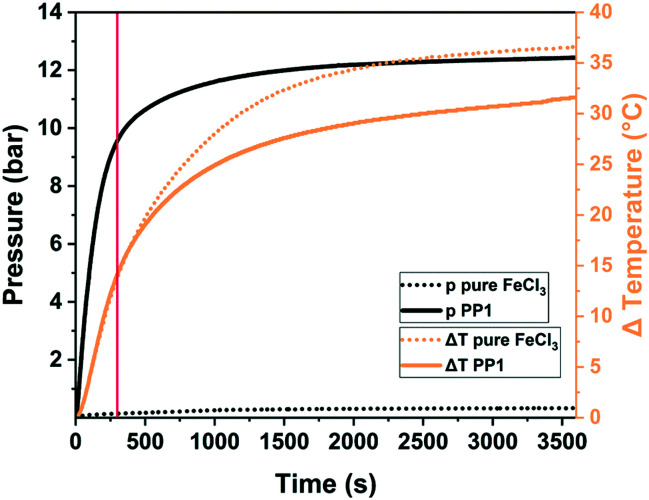
Pressure (solid black) and temperature (solid orange) evolution during the mechanochemical Scholl reaction of 1,3,5-triphenylbenzene (PP1). The pressure and temperature changes for the milling of pure FeCl_3_ are shown as dotted lines. The usual synthesis time of 5 min is marked by a solid red line. Reproduced with permission from ref. 53. Copyright (2020). The Royal Society of Chemistry.

One of the key questions relates to the origin of porosity in the mechanochemical Scholl reaction producing porous polymers. While conventional solution-based syntheses often claim the monomer molecular structure, size, and geometry to be responsible for porosity (molecular design concept), we believe that this is rather unlikely for mechanochemical approaches, particularly for the Scholl reaction. Typically, monomers yielding highly porous polymers in solution do not do so when used in mechanochemical synthesis, and *vice versa*, the porosity of mechanochemically produced polymers can exceed that of the same polymers synthesised in solution. We believe that the reaction kinetics, cross-linking, LAG, and the atmosphere within the milling vessel significantly influence polymerisation and, hence, the porosity of the produced polymer. As mechanochemical reactions are conducted in sealed milling vessels, reactions producing gaseous by-products, *e.g.*, HCl for the Scholl reaction, build up gas pressure in the vessel. Borchardt and coworkers measured a pressure increase of 12 bar using gas pressure and temperature sensors (GTM system) integrated into the vessel cap and found that the emerging HCl is capable of inflating the polymer and is therefore responsible for high SSA_BET_ (Fig. 18).^53^ In this context, LAG, that is the addition of small quantities of a liquid, impacts the mechanochemical Scholl polymerisation in three ways. First, if the liquid is volatile, it can contribute to the build-up of internal pressure; second, if the liquid is halogenated, it can react with FeCl_3_ to form highly reactive intermediates and thus accelerate HCl formation; and third, halogenated liquids can serve as cross-linkers in Friedel–Crafts-type reactions. In any case, the amount of added liquid is very crucial, as revealed by the simultaneous studies of Horike and coworkers and Borchardt and coworkers, who probed the polymerisation of 1,3,5-triphenylbenzene. Both groups synthesised highly porous polymers in as little as 5 min by neat grinding; however, the addition of MeOH led to pore collapse in one case and to increased porosity in the other case,^53,267^ which was ascribed to differences in the amount of used liquid. Whereas the Borchardt group conducted LAG experiments with 1 mL of MeOH (*η* = 0.25 μL mg^−1^), Horike and coworkers determined the optimal amount of MeOH as 0.1–0.3 mL (*η* = 0.03–0.08 μL mg^−1^). This substantiates the enormous importance and sensitivity of LAG in mechanochemical approaches and shows that the liquid additives should not be treated as conventional solvents, but rather as catalysts that exert strong influence in the smallest quantities.

In summary, the mechanochemical Scholl reaction can be carried out over a period as small as 5 min in the absence of large amounts of toxic solvents. The yield and specific surface area of the synthesised polymers are strongly dependent on the amount of Lewis acid, milling frequency, and amount of liquid used for LAG experiments. Special scrutiny, however, has to be paid to the evaluation of the reaction parameters, as even the smallest changes can make the difference between low and high yield and no or high porosity.

### Friedel–Crafts polymerisation

**Fig. 19:**
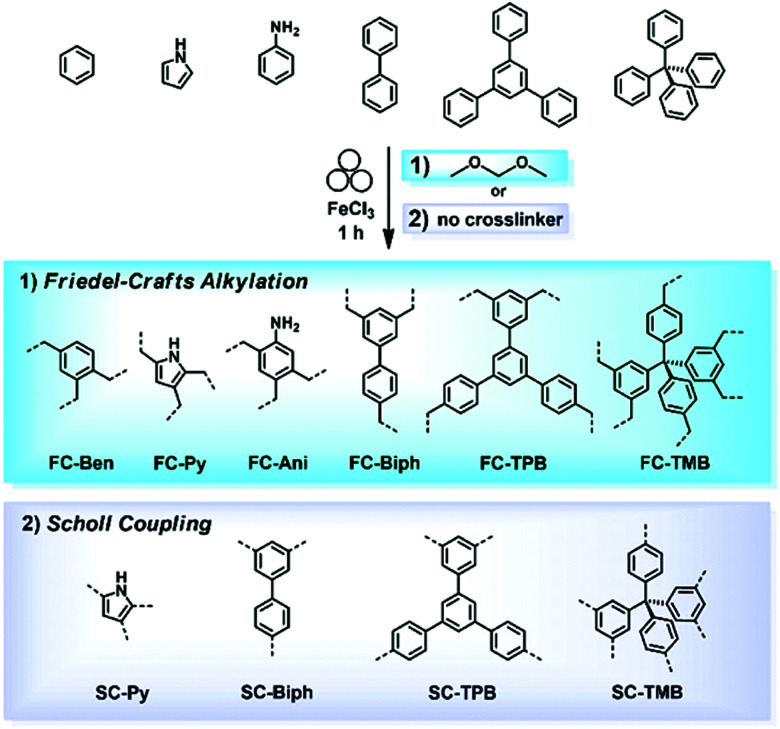
Mechanochemical generation of hyper-cross-linked polymers by (1) Friedel–Crafts alkylation and (2) the Scholl reaction. All products were obtained by ball milling for 1 h in the presence of FeCl_3_ as a Lewis acid. Reprinted with permission from ref. 267. Copyright (2020). The American Chemical Society.

The Friedel–Crafts polymerization is a step-growth polymerization that is driven by a non-directed electrophilic aromatic substitution. Similar to the Scholl polymerization, a catalytic amount of Lewis acid, but in contrast no additional oxidant, is required for the C–C coupling. During the polymerization, the Lewis acid creates an electrophilic reagent (either an external cross-linker or an intramolecular group) that is capable to break the aromaticity of the targeted ring. After the release of a proton, often as HCl, the rearomatization takes place, whereby oligomers are formed that interconnect towards a porous network. Conventional Friedel–Crafts alkylation of porous organic polymers usually requires long heating times of up to 36 h,^268^ gel formation,^269^ or prolonged stirring (up to 48 h) in hazardous solvents such as dichloromethane, chloroform, or 1,2-dichloroethane.^270^ These deficiencies can be circumvented using a mechanochemical approach drastically decreasing the synthesis time down to 35 minutes. Despite the similarities between both reactions, the mechanochemical Friedel–Crafts reaction yields porous polymers with higher surface areas than Scholl coupling under similar conditions.^53,267,271^ While the mechanochemical Scholl reaction of various monomers leads to the formation of conjugated porous polymers, the additional crosslinker induced by the Friedel–Crafts polymerization breaks the conjugation and functions as a spacer between the monomers, allowing a higher flexibility during network formation, accompanied by an enhanced SSA_BET_ of the resulting polymer. Recently, the Borchardt group has compared the Scholl and Friedel–Crafts polymerisations of 1,3,5-triphenylbenzene. While the neat-grinding Scholl reaction yielded a polymer with an SSA_BET_ of 658 m^2^ g^−1^,^53^ the introduction of a cross-linker allowed the synthesis of highly flexible polymers with SSA_BET_ values of up to 1670 m^2^ g^−1^.^271^ Nevertheless, the surface area of polymers generated by the mechanochemical Friedel–Crafts reaction was highly dependent on the monomer building blocks used, as investigated by the group of Horike (Fig. 19).^267^

Friedel–Crafts alkylations are not limited to the utilisation of external cross-linkers, and intramolecular groups can also function as cross-coupling reagents, as presented by Borchardt and coworkers.^89^ In this case, 4,4′-bis(chloromethyl)-1,1′-biphenyl (BCMCP) was milled with anhydrous FeCl_3_ in a planetary ball mill to yield a microporous polymer after 35 min. Upon evaluating the milling parameters, the group identified the differences between mechanochemical Scholl and Friedel–Crafts reactions. Whereas the SSA_BET_ of polymers produced by the Scholl reaction mainly depended on the amount of Lewis acid equivalents, this parameter was of minor importance for the Friedel–Crafts reaction. Similarly, the reaction time hardly influenced polymer porosity. The strongest effect was exerted by grinding frequency.

**Fig. 20:**
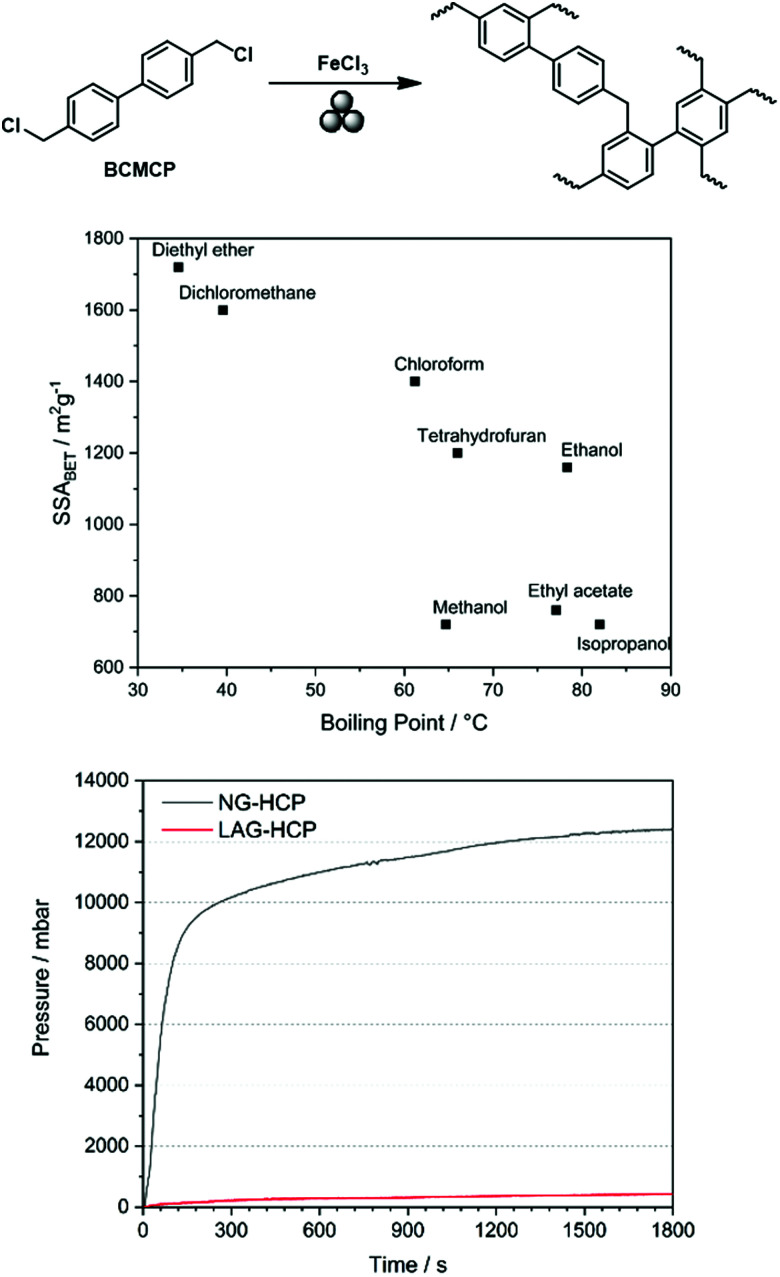
Top: Scheme of mechanochemical Friedel–Crafts alkylation yielding a hyper-cross-linked polymer. Middle: Correlation between the boiling point of the liquid added to the milling jar for LAG and the SSA_BET_ of the generated polymer. Bottom: Pressure development during neat grinding-based (black) and LAG-based (red) syntheses of hyper-cross-linked polymers. Reprinted with permission from ref. 89. Copyright (2019). *Beilstein J. Org. Chem*.

Analogous to the Scholl reaction, excessively low frequencies led to an incomplete reaction and thus to the generation of non-porous polymers or oligomers, while excessively high frequencies caused porosity degradation. Furthermore, SSA_BET_ could be further increased using LAG, as already observed for the Scholl reaction. Initially, the boiling point of the added liquid seemed to be correlated with the obtained specific surface area, which suggested that the liquid might serve as a porogene. Although recent investigations show that this simple correlation does not hold,^53^ the advanced synthesis approach allowed SSA_BET_ to be increased to 1720 m^2^ g^−1^, which exceeded the value of 1450 m^2^ g^−1^ obtained for the solution-based reference (Fig. 20).^89^

In 2019, the group of Zhou proved the versatility of mechanochemical Friedel–Crafts alkylation by generating a hyper-cross-linked polymer from 1,1,2,2-tetraphenylethene as the starting material.^272^ As described above,^89^ anhydrous FeCl_3_ was added to the milling jar to promote cross-coupling, and milling was performed in a planetary ball mill at 500 rpm for 35 min.

**Fig. 21:**
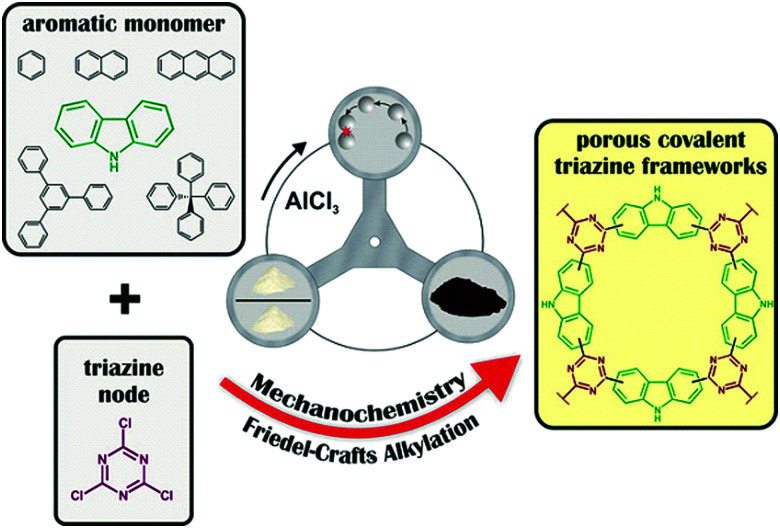
Mechanochemical Friedel–Crafts linkage of various aromatic monomers and a triazine node to yield porous covalent triazine frameworks. Reprinted with permission from ref. 276. Copyright (2017). Wiley-VCH.

The mechanochemical Friedel–Crafts polymerisation can furthermore be used to synthesise covalent triazine frameworks, a class of porous polymers typically produced using complex and time-consuming pathways relying on ionothermal trimerisation^273^ or the utilisation of trifluoromethanesulfonic acid as a catalyst.^274,275^ Mechanochemical synthesis allows many of these issues to be circumvented (Fig. 21).^276^ Borchardt and coworkers linked various monomers such as carbazole, benzene, naphthalene, anthracene, and 1,3,5-triphenylbenzene with a triazine node (cyanuric chloride) in the presence of AlCl_3_ and ZnCl_2_. A yield of >90% was achieved after 1 h of milling for the carbazole system, and surface areas as high as 590 m^2^ g^−1^ were realised.

The characterisation of mechanochemically derived porous polymers is challenging in two ways. First, the reaction is conducted in dense milling vessels rotating or vibrating at frequencies of several hundred times per minute, which precludes the application of most established *in situ* analytical approaches. Fortunately, several groups have developed advanced *in situ* analytics, particularly for milling processes. In principle, these methods are based on *in situ* Raman spectroscopy, *in situ* XRD, and *in situ* temperature/pressure sensing. The latter is a helpful tool to track mechanochemical Friedel–Crafts reactions (Fig. 20 bottom). For the abovementioned reaction, a steep increase in pressure is related to the evolution of HCl gas, and thus, the initiation of the polymerisation reaction. In view of the exothermic nature of polymerisation, a temperature peak at exactly the same time indicates reaction initiation. The second challenge of porous polymer characterisation relates to the insolubility and amorphous structure of these polymers, which precludes characterisation by solution-phase NMR spectroscopy and XRD. Borchardt and coworkers therefore established a comprehensive characterisation method based on dynamic nuclear polarisation (DNP) NMR spectroscopy, which relies on NMR signal amplification by several orders of magnitude *via* the transfer of the electron spin of a polarising agent in the pores of the polymer to the nuclear spin of the polymer backbone atoms.^277^ Thus, DNP-NMR spectroscopy allows one to elucidate the monomer connectivity, possible cross-linkages, and the atomic environment of mechanically produced polymers. Although the whole process is rather expensive and requires a detailed radical screening to be performed prior to analysis, DNP-NMR spectroscopy is a valuable characterisation method for amorphous and insoluble porous polymers that, compared to classical solid-state NMR spectroscopy, is much faster, enables access to multiple heteroatoms, and features a better signal-to-noise ratio.

In summary, although the mechanochemical Friedel–Crafts reaction exhibits disadvantages, such as the formation of HCl, which can corrode the grinding jars and the mill, this solvent-free approach can outperform its solution-based analogue in terms of synthesis time and generated surface area. Furthermore, the above reaction is not as vulnerable to grinding parameter changes as the Scholl reaction and is therefore considerably more reliable for the synthesis of porous polymers. Interestingly, the Friedel–Crafts reaction is suitable not only for the production of hyper-cross-linked polymers, but also for the formation of covalent triazine frameworks.

### Imine, enamine, and amide formation

The formation of imines, enamines, and amides plays a key role in the generation of porous polymers and therefore forms a separate area of mechanochemical polycondensation reactions. Typically, these step-growth polymerisations involve monomers with nitrogen-containing groups, such as amines, and monomers with carbonyl functionalities, such as aldehydes, ketones, or carbonyl chlorides. In contrast to the polymerisation reactions mentioned previously, HCl and water can also be released during condensation. Imine, enamine, and amide formations are commonly used for the synthesis of porous polymers known as covalent organic frameworks (COFs).^278^ These frameworks can either feature 2D (first reported in 2005)^279^ or 3D (first reported in 2007)^280^ topologies and usually contain light elements such as boron, carbon, nitrogen, and oxygen.^280^ In contrast to the aforementioned porous polymer types, COFs exhibit crystalline and well-ordered structures, which require the synthesis under harsh reaction conditions in many cases.^281^

**Fig. 22:**
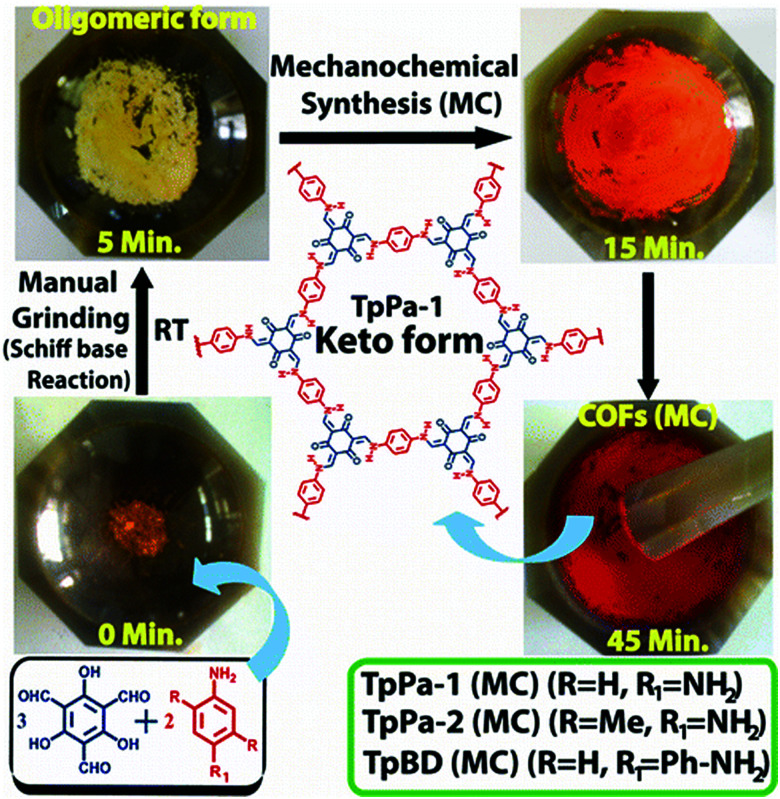
Mechanochemical Schiff-base reaction induced by manual grinding and signified by a colour change from orange over yellow (oligomer) towards red (polymer). Reprinted with permission from ref. 282. Copyright (2013). The American Chemical Society.

To circumvent these issues and develop a fast and simple synthetic procedure, the group of Banerjee showcased the mechanochemical COF formation in their pioneering study in 2013 (Fig. 22).^282^ In this study, three isoreticular COFs were constructed by manual grinding in a mortar-and-pestle setup under ambient conditions. The advantages of this approach are the avoidance of solvents and a drastic decrease in synthesis time from days to 45 min. However, one disadvantage is the need for a long and constant energy input by manual grinding. Although several imines, enamines, and amides have been prepared in a mortar-and-pestle setup,^64,283,284^ a more elegant approach is ball milling,^285,286^ as it guarantees a certain reproducibility and does not rely on the constant energy input of the human operator.

One example is the generation of a bipyridine-functionalised COF in a high-speed ball mill.^287^ For direct comparison, a solvothermal procedure was also established, requiring harsh conditions such as ultrasonication, freeze–pumping, and heating for 72 h in a vacuum-sealed tube. In contrast, the mechanochemical synthesis was accomplished in a rapid (90 min, 30 Hz) and environmentally benign fashion under ambient conditions, circumventing the generation of solvent waste and the use of long heating times.^287^

**Fig. 23:**
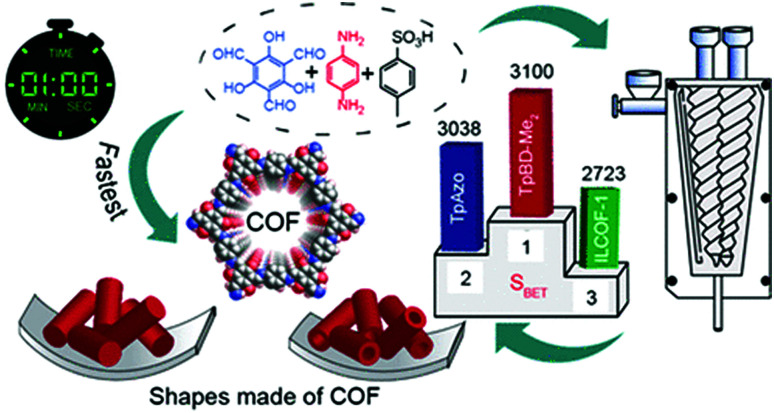
Molecular organisation concept based on the utilisation of PTSA during the mechanochemical reaction. This concept enabled the synthesis of various COFs with high specific surface areas (middle) as well as continuous synthesis (right). Reprinted with permission from ref. 288. Copyright (2017). The American Chemical Society.

A certain disadvantage of ball milling procedures is the limited batch size, which results in low throughput. The need for solvent-free scalability motivated Banerjee to transfer the well-established COF synthesis^282^ to a twin-screw extruder (Fig. 23).^288^

Although this approach might seem questionable, as the COF synthesis relies on reversible covalent bond formation and, hence, on prolonged synthesis times, the introduction of *p*-toluenesulfonic acid (PTSA) enabled molecular organisation and drastically reduced the synthesis time. This concept was used to realise the large-scale (several kg h^−1^) formation of COFs *via* extrusion for 5–10 min followed by heating at 170 °C for 60 s.^288^

The utilization of PTSA during the COF synthesis is not solely important to reduce the long reaction times. The acid furthermore acts as a template to achieve the formation of highly crystalline frameworks. Owing to the random displacement of the mechanochemically exfoliated layers, 2D COFs often exhibit a graphene-like layered structure with low-range crystallinity.^289^ Although this mechanochemical exfoliation is required in certain cases, *e.g.*, for the mechanochemical delamination-based production of covalent organic nanosheets,^290,291^ it generally poses a threat to the synthesis of highly crystalline materials. However, in the presence of PTSA, the protonation of primary amines is followed by the formation of PTSA-amine salts, which act as a template to minimise network defects.

In addition to the template method, the addition of small quantities of liquid was found to have a positive impact on the crystallinity of mechanochemically synthesised COFs.^292^ The mechanochemical reactions of 1,3,5-triformylphloroglucinol and melamine was performed under various conditions, *e.g.*, using neat grinding, LAG, and PTSA-assisted grinding. While neat grinding and ball milling with 1 mL of solvent resulted in the formation of COFs with poor crystallinity, the addition of PTSA or 3 mL of solvent generated COFs exhibiting high crystallinity and a well-defined thread- or ribbon-like morphology, respectively.^292^

Furthermore, the LAG approach facilitated COF formation even in a mortar-and-pestle setup.^284^ The related study identified enol-to-keto tautomerism as a key factor responsible for high stability, which was underlined by the fact that the prolonged water or acid treatment of the β-ketoenamine COF did not affect its crystallinity, while the imine COF was unstable even in a humid environment.^284^

In addition to having low crystallinity, mechanochemically generated COFs often exhibit lower porosity than their classically synthesised counterparts.^287,293^

**Fig. 24:**
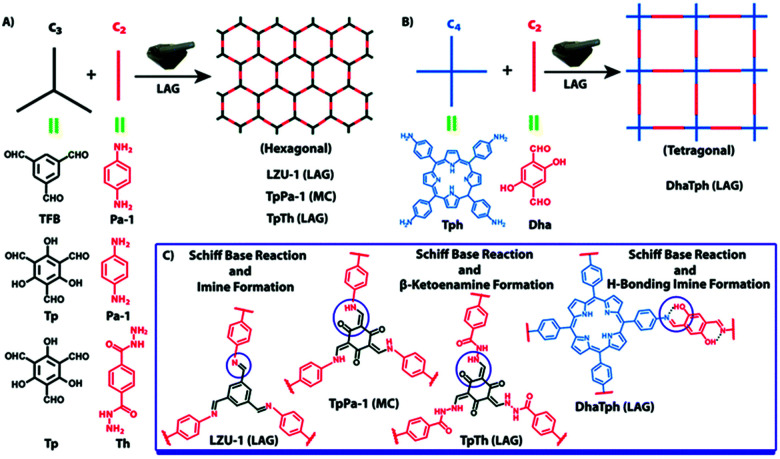
Schematic manual grinding-based LAG synthesis of (A) hexagonal and (B) tetragonal COFs as well as (C) products of the Schiff-base reaction and their respective bonding motifs (blue circles). Reproduced/adapted from ref. 284. Copyright (2014). The Royal Society of Chemistry.

Although the use of LAG increased the crystallinity of the synthesised polymers, this effect was not observed for porosity. The liquid-assisted manual grinding of imine, β-ketoimine, and imine-linked COFs (Fig. 24) resulted in the formation of polymers with low BET surface areas and reversible type II isotherms.^284^ The authors suggested that the mechanochemical synthesis procedure leads to thin-layered structures, hindering a long range pore formation.

Despite this, the addition of PTSA not only drastically increased the crystallinity of the generated COFs, but also had a similar effect on polymer porosity.^288^ The template method enabled a 2–3-fold increase in BET surface area in comparison to the solvothermal methods, which was attributed to long-range porosity and ordered pore channels obtained in the former case.^288^

In addition to this, the group of Dai showed that the structure of the monomer is an important factor to achieve a decent porosity of the resulting polymer by the utilization of pillar[5]quinone, a monomer exhibiting an intrinsic cavity.^294^

Recently, the Borchardt group investigated the mechanochemical synthesis of PI-COFs with a special focus on the “shaking *vs.* baking” question.^199^ Within the two-step condensation reaction, the first step (amic acid formation) was found to proceed readily under ball milling conditions, attributed to the five-ring opening reaction exhibiting a negative enthalpy. However, the second step (imide formation) could solely be achieved by a 30 minutes’ heat treatment, although the Gibbs free energy was found to be negative for the ball milling reaction. The group calculated that the condensation is entropically unfavored, since the generated water is not capable to leave the sealed milling vessel. By a thermal treatment in an open vessel, the water can leave the reaction mixture, shifting the equilibrium towards the imide formation. This result is of high importance as it enables a deeper understanding of the question why some mechanochemical reactions proceed readily, while others require an additional heating step.

In summary, mechanochemical imine, enamine, and amide formations are widely used to generate COFs but are also suitable for the formation of other porous polymers. One of the biggest problems faced by the synthesis of various COFs is their lower crystallinity and porosity compared to those of their counterparts prepared by solution-based synthesis. Furthermore, especially for the synthesis of 2D COFs, special care has to be taken in the parameter evaluation, as already manual grinding might lead to a mechanochemically exfoliation of the layers. Nevertheless, molecular organisation concepts such as PTSA-assisted grinding allow one to minimise network defects and counteract low crystallinities and porosities. Furthermore, these concepts allow reaction upscaling, which renders the entire process highly interesting for the industry. In general, all three mechanochemical polymerisation reactions are sustainable alternatives to classical solvent-based syntheses, allowing the rapid and inexpensive generation of various porous polymers.

### Other polycondensation reactions

In addition to the three main synthetic strategies mentioned above, several other polycondensation-based pathways have been reported.

**Fig. 25:**
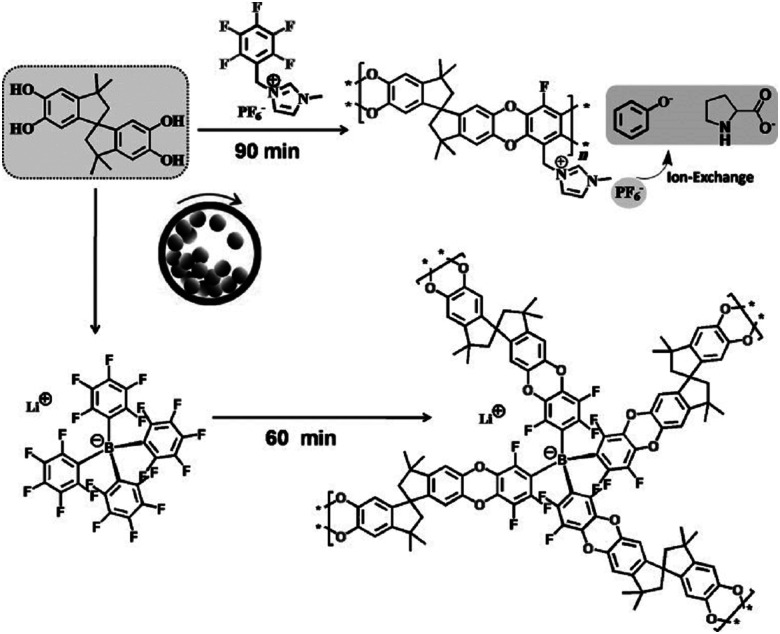
Mechanochemical polycondensation affording charged porous polymers and enabling the synthesis of anionic (bottom) and cationic (top) polymers. Reprinted with permission from ref. 296. Copyright (2015). Wiley-VCH.

In 2015, Dai and coworkers^295^ reported a base-catalysed polymerisation reaction between tetrahydroxy compounds and tetrafluoro compounds induced by high-speed ball milling and affording polymers with oxygen bridges. The obtained polymers of intrinsic microporosity (PIM) showed specific surface areas of >500 m^2^ g^−1^ and featured higher molecular weights and narrower molecular weight distributions than their counterparts prepared in solution, which was attributed to the fast precipitation of the latter in the classical synthesis. The same group transferred this concept of hydroxyl-fluoro-group coupling to charged porous polymers (CPPs) (Fig. 25)^296^ and realised cationic or anionic (depending on the nature of the fluorine-activated ionic monomer) CPPs. It is important to mention that depending on the backbone of the respective CPP, this mechanochemical concept drastically reduces the reaction time (60–90 min) as compared to the solvent-based process (dozens of hours). The cationic CPP polymer was further modified by a simple ion-exchange method to generate CPPs with phenolic or proline counter-anions for capturing and recovering SO_2_ from the atmosphere.

**Fig. 26:**
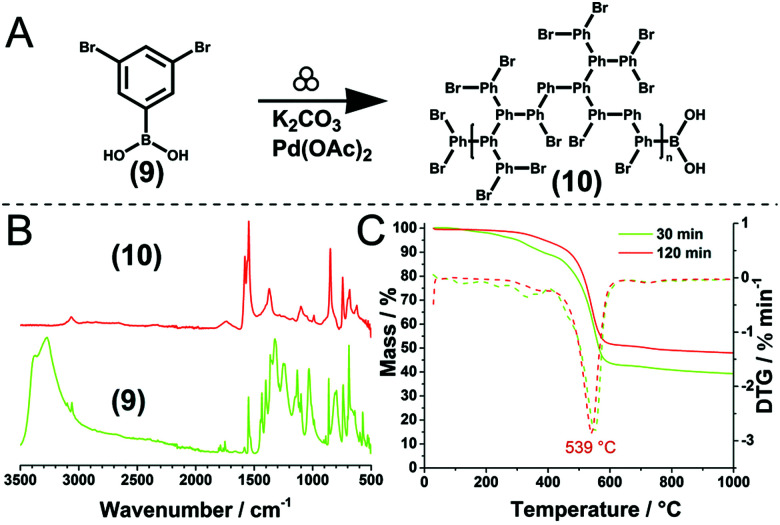
(A) Mechanochemical Suzuki polycondensation affording hyperbranched polyphenylene. (B) Infrared spectra of the monomer (9) and the polymer (10) confirming the polymerisation. (C) Thermal analysis of polymers generated by milling for 30 min (green) and 120 min (orange). Reproduced from ref. 90. Copyright (2017). The Royal Society of Chemistry.

Another concept of porous polymer synthesis relies on cross-coupling reactions. Borchardt and coworkers synthesised hyperbranched polyphenylenes^90^*via* the Suzuki polymerisation of 3,5-dibromophenylboronic acid. The Pd-catalysed reactions of arylboronic acids and aryl halides have been proven to afford linear polyphenylenes. The introduction of branching points (Fig. 26) *via* an A2B1-type monomer provided access to a mesoporous hyperbranched polymer, which was obtained in up to 84% yield and featured a branching degree of 0.74, which is close to the literature value of 0.7.^297^

A recent example is the mechanochemical Ullmann cross-coupling of electron-deficient aromatic monomers containing C–X bonds.^298^ This approach involves the dehalogenation of aryl halides with Mg followed by the formation of a Grignard-type intermediate and thus circumvents the need for electron-rich aromatic building blocks and potential methylene cross-linking, which otherwise limits the aromaticity and conductivity of the porous polymers. As a result, the aromatic ring-knitting reaction enables the solvent-free and straightforward synthesis of prospective electrode materials and illustrates the possibility of using various reaction types for the mechanochemical synthesis of porous polymers.^298^

### Challenges and outlook

Although the mechanochemical generation of porous polymers is a versatile and sustainable alternative to the classical solvent-based approach, it exhibits certain drawbacks. One of the most prominent disadvantages is the degradation of porosity due to prolonged milling times or very high milling frequencies. Additionally, the milling material density and ball size can also have a significant impact in this regard. In the case of excessive energy input due to any of these parameters, the as-synthesised polymers may collapse because of mechanical degradation, which leads to a deterioration of the polymer network, the formation of oligomers, and the associated SSA_BET_ decline. Moreover, these parameters also determine the abrasion degree of the milling material. During mechanochemical synthesis, debris produced by excessive abrasion might not only contaminate the polymer, but also clog its pores to invalidate the results of surface area measurements. Especially for Scholl and Friedel–Crafts polymerisations, one needs to consider the formation of HCl as a by-product, as it may corrode not only the beakers, but also the mill itself. Furthermore, the formation of HCl leads to a large pressure increase inside the milling vessel, which results in the need for appropriately sealed milling beakers and limits the choice of milling devices as well as upscaling possibilities.

Although some of these disadvantages require a longer evaluation of the experimental parameters to be optimised or cannot be avoided at all, the advantages of the mechanochemical synthesis of porous polymers over the classical solvent-based approach generally overweigh the related disadvantages. This is not only due to the significantly shorter reaction times, the avoidance of expensive catalysts and starting materials, and the circumvention of the premature precipitation of polymers from the reaction solution, but also due to the avoidance of large quantities of toxic solvent waste, which drastically enhances the green metrics of the porous polymers (see subsequent section for details).

Until now, only step-growth polymerisation has been used in the synthesis of porous polymers. In general, these approaches are merely a copy of the protocols used in solution. In the future, we believe that hyperbranched polymers will be easily accessed by chain-growth polymerisation. As it has been shown that order and crystallinity are not a prerequisite for the successful application of such materials, the indirect growth of such systems is not a problem. This is especially true because highly ordered structures are difficult to obtain without the reversibility and long reaction times provided by solution syntheses.

However, this is a problem for the characterisation of materials prepared by mechanochemistry, which are generally insoluble, have low crystallinities, and can only be characterised by solid-state methods. However, the latter methods have improved over the years, and access to DNP-NMR spectroscopy or XPS is becoming easier for scientists in all disciplines.

Furthermore, linear and porous polymer communities often use different terminologies and adhere to different standards. As mechanochemical polymerisation seems to readily produce insoluble linear polymers, these communities should come together and establish a common language.

## Green metrics discussion

In the previous chapters, we described the differences between classical and mechanochemical syntheses based on reaction times, on the hazardous potential of the solvents or on the need for a high energy demand due to heating. However, these concepts are relatively vague and a detailed analysis of the underlying principles requires certain standardized parameters. Based on the twelve principles of green chemistry,^299^ a green metrics discussion is a versatile tool for identifying the sustainability of a chemical process,^16,300^ enabling the standardised quantification of the ecological impact of a reaction pathway and providing comparability for a variety of processes according to predefined categories. In the early 1990s, the environmental factor (E-factor) was introduced by Sheldon in response to the growing awareness of waste generation in chemical manufacturing throughout the 1980s.^301,302^ The E-factor is defined as the total waste mass of a process divided by the product mass and therefore accounts for auxiliary chemicals and waste generated by workup.
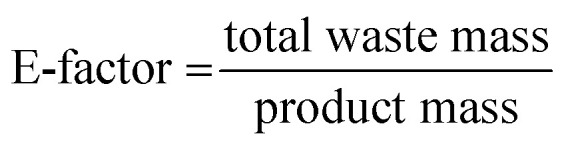
Therefore, a high E-factor indicates an environmentally unfriendly process. However, this factor is strongly dependent on the definition of waste.^302^ Previously, waste was defined as everything not being a product, with the exception of water, so as not to distort the factor. Nevertheless, the disposal or recycling of water might require additional processing steps and should therefore be included in the calculation. As the E-factor can change by orders of magnitude upon water inclusion, even for otherwise harmless processes, calculations are often performed both with and without water.

The second concept, which has been well known since the early 1990s, is the atom economy (AE), which is defined as the molecular weight of the desired product divided by the sum of the molecular weight of all reactants in percent.^303^
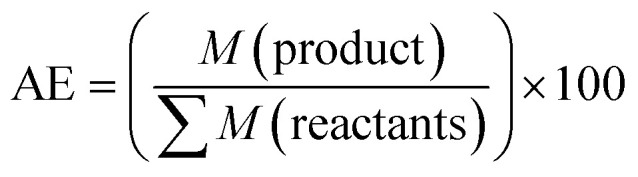
As this factor is based on the stoichiometric quantities of starting materials and products, the amount of solvent is usually not taken into consideration. Therefore, the AE for a mechanochemical and a solvent-based reaction yielding the desired product by the same reaction pathway would be identical, although the latter process would be more environmentally unfavourable because of the generated solvent waste. However, this factor allows the rapid comparison of various synthetic pathways affording a specific product.

Among the more specific parameters related to AE, one should mention carbon efficiency (CE), which is calculated as the amount of carbon in the product divided by the amount of carbon in the reactants in percent.^304,305^ Similarly, the reaction mass efficiency (RME) is also based on the AE, expressing the mass of reactants remaining in the product.^304^ Although both concepts are sufficient for the comparison of various routes towards one product, they do not account for waste and are therefore inadequate for the improvement of process conditions.

To achieve this, the mass intensity (MI) can be determined, which is another very important green chemistry metrics and is defined as the total mass included in the process divided by the mass of the product.^306^
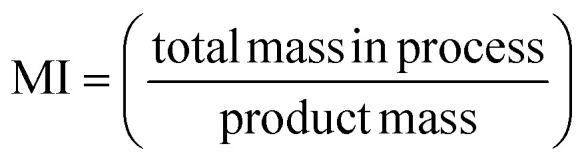
The term therefore includes reagents, solvents, stoichiometry and yields and exhibits a mass ratio depending on all reaction participants. In contrast to the E-factor, which equals zero in the ideal case, a highly sustainable reaction has a mass intensity of unity.^304^ Likewise, for a direct comparison with AE, one can use mass productivity (MP), which is the reciprocal of MI and is expressed in %.
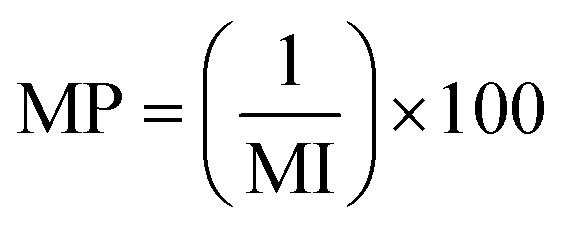
A distinct example for the green metrics discussion of a mechanochemical polymer synthesis was provided in 2017 by Grätz *et al.*,^90^ who discussed the sustainability of a mechanochemical Suzuki polymerisation and directly compared it to that of a solvent-based approach in terms of AE, MI, and MP. For the calculation, the washing procedure was excluded for both processes, as it was assumed to involve equivalent amounts of solvent. Although this assumption allows one to simplify the system, an in-depth analysis for comparison with other processes still requires to consider the mass of all involved chemicals, including those utilised for washing.

During the investigation, the authors calculated the AE to be 38% for each process, as both reaction pathways exhibited the same stoichiometry. Nevertheless, distinct differences were found in the case of MI, which was calculated as 71.4 for the solvent-based approach and as 22.0 for the mechanochemical reaction. By avoiding the use of the bulking material during the mechanochemical reaction, one could decrease the above value to 9.4, which is fairly close to the ideal value of unity and corresponds to a MP of 10.6% that exceeds the solution-based MP by an order of magnitude. In addition, the reaction time was compared, as a shorter process can generally be declared as more favourable. In contrast to the 6 h reaction in solution, mechanochemical Suzuki polymerisation was accomplished within 30 min.

Recently, the same group calculated the green metrics of a mechanochemical Friedel–Crafts polymerisation.^271^ In this study, 1,3,5-triphenylbenzene was cross-linked with either DCM or CHCl_3_ in the presence of the Lewis acid AlCl_3_, which served as the bulk material and the catalyst. The authors provided a detailed calculation of AE, MI, MP, and the E-factor for the solvent-based synthesis, for the mechanochemical synthesis, and for the mechanochemical synthesis without additional bulk material. In the case of no additional bulk material, 6 eq. AlCl_3_ was used to catalyse the reaction. While the solvent-based DCM cross-linking synthesis utilised 20 mL of DCM, the mechanochemical approach only required 0.63 mL to cross-link the monomer. Therefore, the mass productivity increased from 3.18% to 23.47%, which corresponded to E-factors of 30.34 and 2.11 for the solvent-based and for the mechanochemical reaction without bulk material, respectively. For the CHCl_3_-based cross-linking, the reduction in solvent volume from 20 mL to 0.78 mL led to a decrease in the E-factor from 35.76 (solvent-based synthesis) to 1.87 (mechanochemical synthesis without bulk material). As the E-factor equals zero in the ideal case, the mechanochemical approach offers an opportunity to drastically decrease the environmental impact of the reaction and provides further important benefits. Whereas the solvent-based synthesis required a time of 48 h and was followed by a 24 h Soxhlet extraction in 100 mL MeOH and 100 mL CHCl_3_, the mechanochemical reaction was accomplished within 30 min and was followed by a short rinse of the polymer in 100 mL H_2_O and 100 mL acetone. Although the mechanochemical synthesis drastically reduced the environmental impact as well as the synthesis/workup time and the toxicity of the liquids required for workup, the resulting porous polymer outperformed its solvent-based equivalent in terms of SSA_BET_ and yield.^271^

Exemplary for other mechanochemical polymerization reactions, the Suzuki and the Friedel–Crafts polymerization can therefore be considered as extremely advantageous in comparison to the solvent-based approach, since the avoidance of solvent waste drastically increases the overall sustainability and the time reduction further promotes energy efficiency as well as cost reduction.^90,271^

Although mechanochemical polymerisations can generally be declared as more environmentally benign than their solution-based counterparts, other synthetic methods may even exceed the sustainability of mechanochemical procedures. Examples of these are reaction pathways that do not require solvents or bulking agents, *e.g.*, polymerisation from the melt.^307^ Nevertheless, such reactions often demand prolonged heating times, which has a negative impact on the overall energy consumption and must be taken into consideration.

**Fig. 27:**
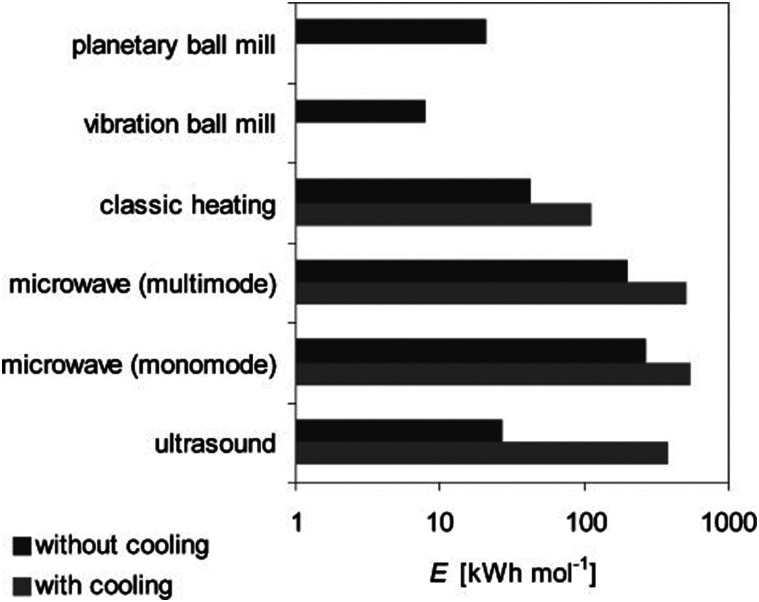
Comparison of the energy consumptions of different processes without cooling (dark grey) and with cooling (light grey). Reprinted from ref. 308. Copyright (2010). Wiley-VCH.

To quantify the energy consumption of ball milling procedures and compare it to that of other prominent methodologies, Stolle *et al.* determined the dependence of molar energies on batch size and line power consumption (Fig. 27).^22,308^ Using various process data, the authors concluded that the energy efficiency of a vibrational ball mill exceeds that of a planetary ball mill, whereas ultrasound and microwave methods are the least efficient.^22^ These results render ball milling procedures well suited for the efficient processing of polymers to enhance the overall sustainability.

Although mechanochemical polymerisations can generally be declared as sustainable and sufficient alternatives to solution-based analogues, which is attributed to the avoidance of solvent waste and the circumvention of mass transport limitations and insufficient energy transfer, it is essential to consider the characteristics of the generated material. Most polymers exhibit excellent corrosion resistance with an attractive cost-to-performance ratio,^309^ which results in large-scale production and environmental pollution due to polymer persistence. In addition to the sustainable generation of polymers, the use of renewable resources and effective waste recycling are important factors for a green and sustainable future.

## Conclusion

The International Union of Pure and Applied Chemistry (IUPAC) announced ‘mechanochemistry’ as one of 10 chemical innovations expected to change the world. While classical syntheses rely on the addition of solvents, mechanochemistry induces reactions through mechanical forces, enabling sustainable syntheses by avoiding large amounts of waste. In particular, this approach greatly benefits polymer synthesis, allowing one to bypass the need for solubilising groups, to avoid low polymerisation degrees due to rapid precipitation, and to access new innovative structures. Nevertheless, mechanochemistry is still widely unknown to chemists in general and, although extremely powerful, is very rarely considered as a tool for polymer synthesis. This review provides insights into and an understanding of constructive mechanochemical polymer synthesis by summarising examples of various mechanochemical polymerisations with higher yields, shorter reaction times, and improved green chemistry metrics compared to those achieved in classical synthetic routes.

## Conflicts of interest

There are no conflicts to declare.

## Supplementary Material
